# Integrated transcriptomic and proteomic profiling reveals the key molecular signatures of brain endothelial reperfusion injury

**DOI:** 10.1111/cns.14483

**Published:** 2023-10-03

**Authors:** Yabin Ji, Yiman Chen, Xixi Tan, Xiaowen Huang, Qiang Gao, Yinzhong Ma, Shilun Yang, Meifang Yin, Min Yu, Cheng Fang, Yu Wang, Zhu Shi, Junlei Chang

**Affiliations:** ^1^ Department of Neurology Nanfang Hospital, Southern Medical University Guangzhou China; ^2^ Institute of Biomedicine and Biotechnology, Shenzhen Institute of Advanced Technology, Chinese Academy of Sciences Shenzhen China; ^3^ Department of Neurology Yangjiang People's Hospital Yangjiang China; ^4^ Department of Neurosurgery The First Affiliated Hospital of Zhengzhou University, Zhengzhou University Zhengzhou China; ^5^ State Key Laboratory of Pharmaceutical Biotechnology and Department of Pharmacology and Pharmacy, Li Ka Shing Faculty of Medicine The University of Hong Kong Hong Kong China; ^6^ Department of Neurology 10th Affiliated Hospital, Southern Medical University (Dongguan People's Hospital) Dongguan China

**Keywords:** blood–brain barrier, inflammation, ischemic stroke, multi‐omics, reperfusion injury

## Abstract

**Background:**

Reperfusion therapy after ischemic stroke often causes brain microvascular injury. However, the underlying mechanisms are unclear.

**Methods:**

Transcriptomic and proteomic analyses were performed on human cerebral microvascular endothelial cells following oxygen–glucose deprivation (OGD) or OGD plus recovery (OGD/R) to identify molecules and signaling pathways dysregulated by reperfusion. Major findings were further validated in a mouse model of cerebral ischemia and reperfusion.

**Results:**

Transcriptomic analysis identified 390 differentially expressed genes (DEGs) between the OGD/R and OGD group. Pathway analysis indicated that these genes were mostly associated with inflammation, including the TNF signaling pathway, TGF‐β signaling pathway, cytokine–cytokine receptor interaction, NOD‐like receptor signaling pathway, and NF‐κB signaling pathway. Proteomic analysis identified 201 differentially expressed proteins (DEPs), which were primarily associated with extracellular matrix destruction and remodeling, impairment of endothelial transport function, and inflammatory responses. Six genes (*DUSP1*, *JUNB*, *NFKBIA*, *NR4A1*, *SERPINE1*, and *THBS1*) were upregulated by OGD/R at both the mRNA and protein levels. In mice with cerebral ischemia and reperfusion, brain TNF signaling pathway was activated by reperfusion, and inhibiting TNF‐α with adalimumab significantly attenuated reperfusion‐induced brain endothelial inflammation. In addition, the protein level of THBS1 was substantially upregulated upon reperfusion in brain endothelial cells and the peri‐endothelial area in mice receiving cerebral ischemia.

**Conclusion:**

Our study reveals the key molecular signatures of brain endothelial reperfusion injury and provides potential therapeutic targets for the treatment of brain microvascular injury after reperfusion therapy in ischemic stroke.

## Graphical abstract

Transcriptomic analysis of human cerebral microvascular endothelial cell (HCMEC)/D3 cells subjected to OGD/R showed that the top significantly changed signaling pathways were mostly associated with inflammatory responses, especially the TNF signaling pathway. Reperfusion‐induced differentially expressed proteins were primarily involved in extracellular matrix destruction and remodeling, impairment of endothelial transport function, and inflammatory responses.

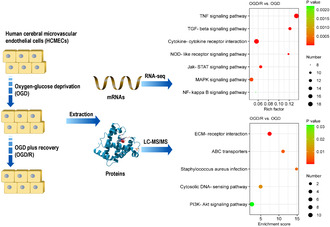

## 1 INTRODUCTION

Ischemic stroke (IS), one of the major diseases that endangers the health and life of elderly individuals, is characterized by high incidence, high mortality, and high rate of disability. The principal treatment of acute IS is to restore the blood supply to the ischemic brain tissue as soon as possible and reduce damage to the penumbra brain tissue. Thrombolysis and intravascular therapy are the main clinical treatment methods for acute IS.^1^, ^2^ However, after a period of ischemia, blood reperfusion may also cause damage to the brain, that is, reperfusion injury,^3^ although the mechanisms of reperfusion injury are not well understood. Microvascular injury and blood–brain barrier (BBB) disruption are the main pathological causes of reperfusion injury, which are mainly manifested as hemorrhage transformation, brain edema, and no‐reflow phenomenon in the infarct area.^4^, ^5^, ^6^, ^7^ These complications not only limit the time window for reperfusion therapy, but also affects the treatment efficacy.

The BBB function is determined by the neurovascular unit that is composed of cerebral vascular endothelial cells, pericytes, astrocyte endfeet, and the surrounding basement membrane. BBB is essential for maintaining the homeostasis of brain tissue microenvironment, regulating brain metabolism, and facilitating the normal neuronal function.^8^ Endothelial cells (ECs) as the main component of the brain vasculature and BBB are the primary target of reperfusion injury. Therefore, a systematic study of the dysregulated molecules and signaling pathways in brain ECs after cerebral ischemia and reperfusion is fundamental to elucidating the mechanisms of reperfusion injury.

In the current study, we performed transcriptomic analysis by RNA‐sequencing (RNA‐seq) and proteomic analysis by liquid chromatography–tandem mass spectrometry (LC–MS/MS) with human cerebral microvascular endothelial cells (HCMECs) following oxygen–glucose deprivation and recovery (OGD/R). The major findings of the in vitro studies were further validated in a mouse model of cerebral ischemia and reperfusion in vivo. Our data provide a comprehensive molecular atlas of brain EC injury induced by ischemia/reperfusion, which will facilitate the understanding of reperfusion injury and exploring of novel therapeutic approaches by targeting ECs after acute IS.

## 2 MATERIALS AND METHODS

### 2.1 Cell line and in vitro OGD/R model

We purchased an immortalized HCMEC line (HCMEC/D3) from Institut Cochin (Paris, France) and cultured it in Dulbecco's modified Eagle's medium (DMEM) containing 10% fetal bovine serum and 1% penicillin. All cultures were maintained in a humidified 5% CO_2_ incubator at 37°C and routinely passaged three times after reaching 80%–90% confluency. The cells were divided into a normal control (NC) group, an OGD‐6 h group, and different oxygen and glucose recovery (OGD/R) time groups (observation time points: 2.5, 3, 4, 5, 7, 14, and 26 h). Endothelial cell activity decreased by >30% in the OGD‐6 h group, which was in line with the requirements of follow‐up studies; setting the peak point of cell damage after OGD/R for follow‐up studies was conducive to comparing the differences between reperfusion injury and ischemia‐hypoxia injury at the molecular level. Three biological replicates were performed for each group of cells, and each biological replicate was performed in three wells. For OGD modeling, the cells were washed three times with phosphate‐buffered saline (PBS) to ensure that no residual sugar remained. The cells were then resuspended in DMEM (Solarbio) and placed in an OGD incubator (99% N_2_) for 6 h. For OGD/R modeling, the cells were removed from the OGD incubator and placed in a conventional oxygen concentration incubator. The sugar/serum‐free culture medium was replaced with complete DMEM, and the samples were taken at the predetermined time point for subsequent experiments. Cell Counting Kit‐8 (CCK‐8; TransGen Biotech) was used to measure cell viabilities, according to the manufacturer's instructions. Briefly, cells were seeded in 96‐well plates at a density of 5000 cells/well. Ten microliters of CCK‐8 solution were added to each well containing 100 μL of medium. The cells were cultured at 37°C for 2 h. Optical density values were measured at 450 nm using Multiskan GO spectrophotometer (Thermo Scientific).

### 2.2 RNA‐seq

#### 2.2.1 RNA extraction and 3′‐end sequencing for expression quantification (3′‐seq)

Total RNA was extracted using the Direct‐zol RNA MiniPrep Kit (Zymo Research). To quantify mRNA expression levels, 3′‐seq was performed as previously described.^9^ Briefly, eukaryotic mRNA was first enriched by Oligo (dT) beads, while prokaryotic mRNA was enriched by removing rRNA using the Ribo‐Zero™ Magnetic Kit (Epicenter). The mRNA was then fragmented into short fragments and reverse transcribed into complementary DNA (cDNA) with random primers. Second‐strand cDNA was synthesized using DNA polymerase I, RNase H, and dNTPs. Then, the cDNA fragments were purified with a QiaQuick PCR Extraction Kit (Qiagen), end‐repaired, modified by poly (A) addition, and ligated to sequencing adapters (Illumina). The ligation products were size‐selected by agarose gel electrophoresis, amplified by the polymerase chain reaction (PCR), and sequenced using a Hiseq PE150 System (Illumina).

#### 2.2.2 RNA‐seq data analysis

The raw RNA‐seq data were subjected to quality‐control analysis (to filter the data and reduce noise) before conducting bioinformatics analysis. Analysis showed that high‐quality reads accounted for >98.95% of all reads in each sample (Table S1). Then, short read alignment tool Bowtie2 (version 2.2.9) was used for mapping reads to the ribosome RNA (rRNA) database. Less than 4.20% of reads in each sample (<1.29% in the treatment groups) matched with the rRNA database (Table S2). The rRNA mapped reads were then removed. The remaining reads were mapped to the Ensembl human genome (GRCh38.p13) using TopHat2 (version 2.1.1) and then assembled using Cufflinks (version 2.2.1). At least 85.15% of the sequences were well aligned in each sample (Table S3). Then, the gene expression levels were normalized using the fragments per kilobase of transcripts per million mapped reads (FPKM) method and the edgeR package (version 3.14.0) was utilized to identify the DEGs across groups. DEGs were defined as those showing a fold‐change (FC) in expression of >2.0 and an adjusted *p* value for the false‐discovery rate (FDR) of <0.05. The Database for Annotation, Visualization, and Integrated Discovery (DAVID) was used to analyze gene abundances. DAVID is a bioinformatics resource that is used to explain the functions of numerous genes. A list of DEGs from the above group comparisons was uploaded to DAVID (version 6.7) to identify enriched biological functions, such as Gene Ontology (GO) terms and Kyoto Encyclopedia of Genes and Genomes (KEGG) pathways. The Gene Set Enrichment Analysis (GSEA) was performed using the gseKEGG function from the R package clusterProfiler (v4.6.0). The enrichment scores were normalized by gene set size, and their statistical significance was assessed by permutation tests (*n* = 1000). The adjust *p* value <0.05 were used as the cut‐off for significance in all GSEA analysis.

### 2.3 Proteomics

#### 2.3.1 Sample preparation

Fifty micrograms of protein were digested in 8 M urea buffer (8 M urea (U5378, Sigma‐Aldrich), 100 mM Tris 8.5 (15,504,020, Thermo Scientific), 10 mM dithiothreitol (D9779, Sigma‐Aldrich)) using the filter‐aided sample preparation (FASP) protocol. Briefly, proteins were added to a 30 kDa cut‐off filter (MRCF0R030, Millipore) and centrifuged at 11,000 rotations/min at 20°C for 15 min. Iodoacetamide (I1149, Sigma‐Aldrich) (50 mM) in urea buffer was used to alkylate the proteins at 20°C for 15 min. After a few washes with urea buffer and 50 mM ammonium bicarbonate (ABC; 09830, Sigma‐Aldrich) buffer, 100 ng of trypsin (V5280, Promega) in 50 mM ABC buffer was used to digest proteins in a wet chamber overnight at 37°C. Peptides were extracted using 50 mM ABC buffer and acidified with trifluoroacetic acid (TFA; 1,082,620,100, Millipore). After performing the FASP protocol described above, the peptides were separated into 5 fractions (flow through, pH 11, pH 8, pH 5, pH 2). Each sample was measured by LC–MS/MS using a 4 h gradient.

#### 2.3.2 Proteomic analysis

Tryptic peptides were separated over 240 min using an Easy‐nLC 1200 ultra‐high‐performance LC system, using the following gradient: 2%–7% buffer B (80% acetonitrile (1,000,041,001, Millipore) in 0.1% TFA) over 11 min, 7%–28% buffer B over 200 min, and 28%–36% buffer B over 15 min. Subsequently, the peptides were washed for 5 min while increasing buffer B from 36% to 60% and for 9 min while increasing buffer to 95%. The Easy‐nLC 1200 system was connected online to a Fusion Lumos mass spectrometer equipped with a FAMIS Pro interface (Thermo Scientific). Scans were collected in a data‐dependent top‐speed mode with a dynamic exclusion of 90 s. Raw data were analyzed using MaxQuant software (version 1.6.0.1) by searching against the Human Fasta Database, with label‐free quantification and matches between run functions enabled. The output protein list was analyzed and visualized using the DEP package.

### 2.4 Middle cerebral artery occlusion/reperfusion (MCAO/R) model and TNF‐α neutralizing monoclonal antibody (mAb) treatment

Male 8–10‐week‐old C57BL/6 mice, weighing 20–23 g, were obtained from Beijing Vital River Laboratory Animal Technologies Co., Ltd. Because menstrual period in female animals causes significant changes in estrogen levels and variations in infarct size and neurological outcome, only male mice were used in this study. Animals were housed in a pathogen‐free animal facility with 12‐h light–dark cycles. All procedures performed on mice were approved and carried out in accordance with the Animal Care and Use Committee of the Shenzhen Institute of Advanced Technology, Chinese Academy of Sciences. Randomized animals were used for the sham‐operation and IS models. For the surgical procedures, anesthesia was induced with 4% isoflurane within air in an induction chamber; anesthesia was maintained with 2% isoflurane within air delivered through a face mask (RWD Life Science). A heating pad was used to maintain each mouse's core temperature at 37 ± 0.5°C throughout the surgical procedure. A modified intraluminal‐filament model was used to induce middle cerebral artery occlusion (MCAO). After 60 min of MCAO, reperfusion was established by retracting the filament. The animals had free access to food and water throughout the reperfusion period. To verify the success of ischemia and reperfusion during MCAO surgery, a laser Doppler flowmetry (PERIMED AB) was used to measure the cortical blood flow supplied by the MCA. Mice in which the cerebral blood flow decreased to levels at least 30% below the baseline value after filament insertion were included in the study. The success of reperfusion was verified by the increase of cerebral blood flow to over 70% of the baseline value after filament retraction. The animals were divided into two different reperfusion groups (6 and 24 h reperfusion periods; samples were taken at a predetermined time point), a control group (samples taken 1 h after inducing MCAO), and a sham‐operation group (*n* = 3–4 mice per group). TNF‐α mAb Adalimumab (Abbott, US; 3 mg/kg) or control human IgG (#SP001, Solarbio) was administered by retro‐orbital i.v. injection immediately after MCAO at the onset of reperfusion. Neurological deficit scores were evaluated after MCAO by an investigator who was blind to the grouping according to a 5‐point Bederson scale: 0, normal motor function; 1, flexion of the torso and the contralateral forelimb upon lifting by the tail; 2, circling to the ipsilateral side but normal posture at rest; 3, leaning to the ipsilateral side at rest; 4, unable to walk spontaneously. Animals were excluded if no neurological deficits were present after surgery, according to the pre‐established exclusion plan.

### 2.5 Infarct size analysis

The brains were sectioned into 2‐mm‐thick sections and placed in 1% 2,3,5‐triphenyltetra‐zolium chloride (TTC; #T8877, Sigma‐Aldrich) for 10 min at 37°C. The TTC solution was then replaced with 4% paraformaldehyde, and the sections were incubated at room temperature for 1 h. The sections were photographed with a digital camera, and the infarct sizes were measured using ImageJ software (National Institutes of Health) by an investigator who was blind to the grouping. The contribution of post‐ischemic edema to the volumes of sites of injury was corrected as described previously.^10^ The infarct size (%) was calculated as ([volume of the left hemisphere – non‐infarct volume of the right hemisphere] / volume of the left hemisphere) × 100%.

### 2.6 Reverse transcriptase‐quantitative polymerase chain reaction (RT‐qPCR)

Total RNA was extracted using the Direct‐zol RNA MiniPrep Kit (Zymo Research). The RNA was reverse transcribed using iScript Reverse Transcription Supermix for RT‐qPCR according to the manufacturer's instructions (Vazyme). The primer sequences are shown in Table S4. RT‐qPCR was performed in a StepOnePlus Real‐Time PCR System using Power SYBR Green (Roche). Relative RNA‐expression levels were calculated using the comparative Ct method and normalized to actin mRNA expression. Data are expressed relative to a calibrator using the 2^−ΔΔCt^ method.

### 2.7 Immunofluorescence staining

Frozen sections (10 μm thick) were allowed to dry on adhesion microscope slides (Citotest Scientific Co., Ltd.) at room temperature and then rehydrated in PBS. The sections were blocked in 10% normal goat serum (Thermo Fisher) in PBS + 0.2% Triton X‐100 for 1 h at room temperature. The samples were incubated at 4°C with the following primary antibodies in PBS + 5% goat serum +0.2% Triton X‐100: hamster anti‐mouse CD31 (1:100, #MAB1398Z, Millipore), donkey anti‐mouse IgG (1:200, #715‐545‐150, Jackson ImmunoResearch), mouse anti‐TNF‐α (1:100, #sc‐52,746, Santa Cruz Biotechnology), rabbit anti‐IL‐6 (1:100, #K009385P, Solarbio), rabbit anti‐CXCL3 (1:100, #orb13448, Biorbyt), rabbit anti‐THBS1 (1:100, #18304‐1‐AP, Proteintech), rabbit polyclonal anti‐PIGT (1:250, #16906‐1‐AP, Proteintech), rabbit anti‐NF‐κB p65 (1:100, #8242, CST, MA), and rabbit anti‐VCAM‐1 (1:100, #39036S, CST, MA). Excess antibody was removed by rinsing three times with PBS (7 min/wash step). Samples were then incubated at room temperature for 1 h with the following secondary fluorescently labeled antibodies from Jackson ImmunoResearch: Cy3 goat anti‐hamster IgG (1:500, #127‐165‐099), Alexa Fluor 488 donkey anti‐mouse IgG (1:500, #715‐545‐150), and Alexa Fluor 488 donkey anti‐rabbit IgG (1:500, #711–545‐152), diluted in PBS + 10% goat serum +0.2% Triton X‐100. Excess antibody was removed by rinsing three times with PBS (5 min/wash step). Slides were mounted in antifade mounting medium with DAPI (Solarbio) and imaged with an Olympus microscope to obtain 20× or 40× images. The immunofluorescence signal area or density was quantified using ImageJ software and normalized according to the vessel area (CD31‐positive area) in three random ischemic areas per mouse.

### 2.8 Western blotting

The protein lysate samples were boiled at 95°C, followed by their separation on polyacrylamide gels ranging from 6% to 12%. Subsequently, the separated proteins were transferred onto PVDF membranes through electrophoresis. To block the membranes, a mixture of 5% non‐fat milk and 0.05% Tween 20 in tris‐buffered saline was used for an hour. The next step involved washing the membranes and then incubating them at 4°C with primary antibodies: rabbit polyclonal anti‐PIGT (1:1000, Proteintech, #16906‐1‐AP), rabbit polyclonal anti‐THBS‐1 (1:1000, Proteintech, #16906‐1‐lg), mouse monoclonal anti‐β‐actin (1:2000, Proteintech, #66009‐1‐AP). For the subsequent incubation step at room temperature, horseradish peroxidase (HRP)‐conjugated secondary antibodies, including anti‐rabbit IgG (Cell Signaling Technology, #7074) or anti‐mouse IgG (Cell Signaling Technology, #7076), were employed. Finally, the immunoreactive bands were detected using enhanced chemiluminescence (ECL).

### 2.9 Statistical analysis

Statistical analysis was performed in Graphpad Prism software (ver. 7). Data are expressed as the mean ± standard error of mean (SEM), and the number (n) of the samples employed is indicated in the figure legends. The Shapiro–Wilk test was used to assess the normality of the data distribution. Statistical differences among multiple groups were compared using one‐way analysis of variance (ANOVA), followed by Tukey's multiple‐comparisons test, while two groups were analyzed by 2‐tailed Unpaired Student's *t*‐test. Statistical significance was defined as *p* < 0.05. The RNA‐seq data were assessed by a bioinformatics team at Sagene Biotech Co., Ltd., and proteomic analysis was performed by a bioinformatics team at LumingBio Company.

## 3 RESULTS

### 3.1 Transcriptomic analysis of HCMECs subjected to OGD/R

The HCMEC/D3 cells were divided into NC, OGD‐6 h, and OGD/R groups and subjected to corresponding treatments. We observed that cells showed the most critical injury at 3 h reperfusion following 6 h of OGD, as determined by assessing cell viabilities in CCK‐8 tests (Figure S1). Therefore, we chose the 3 h‐OGD/R as a representative time point for reperfusion injury in vitro.

**FIGURE 1:**
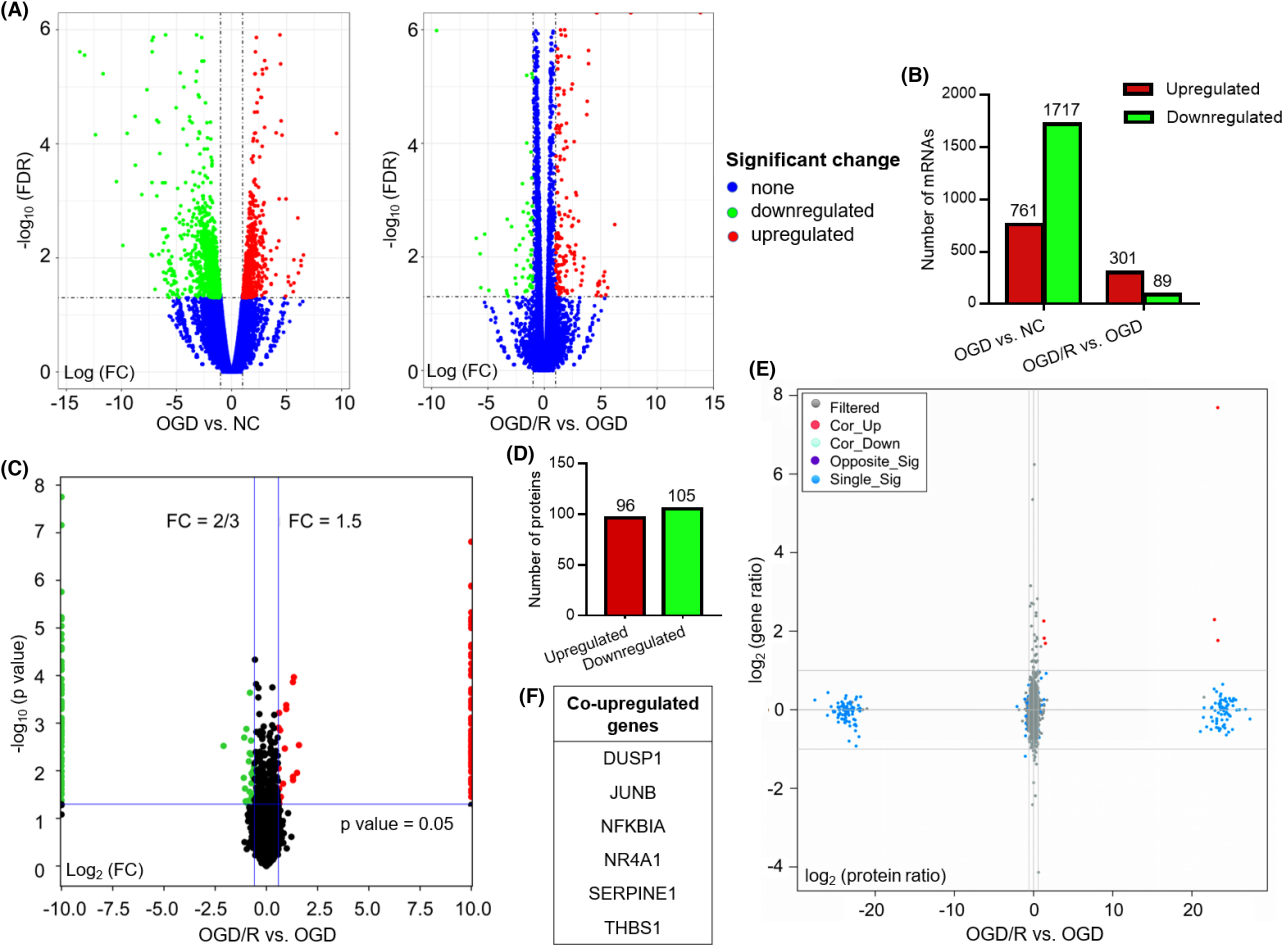
Integrative transcriptomic and proteomic analysis of HCMEC/D3 cells subjected to OGD/R. (A) Volcano plots of DEGs for the transcriptomic analysis. Red dots represent upregulated genes, and green dots represent downregulated genes. (B) Bar graph demonstrating the number of upregulated and downregulated genes. DEGs were defined as those with an FDR of <0.05 and an absolute FC of >2.0. (C) Volcano plots of DEPs for the proteomic analysis. Red dots represent upregulated proteins, and green dots represent downregulated proteins. (D) Bar graph demonstrating the number of upregulated and downregulated proteins. DEPs were defined as those with a *p* value of <0.05 and an absolute FC of ≥1.5. (E) Quadrant map of DEGs and DEPs. The red dots represent co‐upregulated mRNAs and proteins, and the green dots represent co‐downregulated mRNAs and proteins. (F) An alphabetical list of co‐upregulated genes at both the mRNA and protein level. DEG, differentially expressed gene; DEP, differentially expressed protein; FDR, false‐discovery rate; HCMEC/D3, a type of human cerebral microvascular endothelial cells; NC, normal control; OGD/R, oxygen–glucose deprivation and recovery.

We used FDR of <0.05 and FC of >2.0 as thresholds for assigning significant differences. The results showed that ECs exhibited different transcriptomic characteristics during OGD and OGD/R. Compared to the NC group, the OGD group had 2478 DEGs with 761 upregulated genes and 1717 downregulated genes. Compared to the OGD, the OGD/R group had 390 DEGs with 301 upregulated and 89 downregulated genes (Figure 1A,B; Data S1).

DEGs‐based KEGG analysis showed that 277, 177, and 268 signaling pathways were associated with DEGs when comparing the OGD and NC groups, the OGD/R and OGD groups, and the OGD/R and NC groups, respectively; among them, 13, 23, and 12 signaling pathways were significantly enriched (Q value ≤0.05; Data S2). The top five pathway terms associated with DEGs between the OGD/R and OGD groups were the TNF signaling pathway, TGF‐β signaling pathway, cytokine–cytokine receptor interaction, NOD‐like receptor signaling pathway, and Salmonella infection (Table 1). This suggests that dysregulation of these signaling pathways (especially upregulation of the TNF signaling pathway, evidenced by upregulation of *TNF*, *IL‐6*, *LIF*, *CXCL1*, *CXCL2*, *CXCL3*, *CCL20*, *NFKBIA*, *TNFAIP3*, *SOCS3*, *MAP2K3*, and other genes; Table 1; Table S5) may play important roles in endothelial reperfusion injury. Furthermore, significantly enriched signaling pathways between the OGD/R and OGD groups were mostly associated with the inflammatory response, including the TNF signaling pathway, TGF‐β signaling pathway, cytokine–cytokine receptor interaction, NOD‐like receptor signaling pathway, JAK–STAT signaling pathway, MAPK signaling pathway, and NF‐κB signaling pathway (Table 1). We further performed whole transcriptome rank‐based GSEA between the OGD/R group and the OGD group. We observed enrichment in 17 distinct KEGG signaling pathways in OGD/R group compared with OGD group (*p*.adjust <0.05; Figure S2 and Data S3). Consistent with the DEGs‐based KEGG analysis, we observed significant enrichment in inflammatory responses, such as IL‐17 signaling pathway, C‐type lectin receptor signaling pathway, cytokine–cytokine receptor interaction, TNF signaling pathway, JAK–STAT signaling pathway, TGF‐β signaling pathway, and chemokine signaling pathway (Figure S2). These results collectively suggest that endothelial cell‐mediated inflammatory responses are the most prominent mechanism underlying reperfusion injury at the mRNA level.

**TABLE 1:** Top ten pathways associated with DEGs in HCMEC/D3 cells in the OGD/R group, as compared with those in the OGD group.

Pathway	Pathway ID	Overlap	*p* value	Q value	Associated genes
TNF signaling pathway	ko04668	19/142	0.000000	0.000000	(Up) *TNF, IL‐6, LIF, CXCL1, CXCL2, CXCL3, CCL20, JUNB, JUN, FOS, EDN1, NFKBIA, TNFAIP3, SOCS3, PTGS2, CSF2, MAP2K3, TCONS_00118147, TCONS_00266386*
TGF‐beta signaling pathway	ko04350	12/99	0.000000	0.000005	(Up) *THBS1, FST, NOG, TNF, GDF6, MYC, INHBA, ID1, ID2, ID3, ID4* (Down) *INHBE*
Cytokine–cytokine receptor interaction	ko04060	19/334	0.000001	0.000086	(Up) *IL‐6, IL‐8, IL‐11, IL‐24, CXCL1, CXCL2, CXCL3, CCL20, CXCR5, CLCF1, TNF, LIF, CSF2, GDF6, INHBA, TNFSF15, PDFGRB* (Down) *KIT, INHBE*
NOD‐like receptor signaling pathway	ko04621	9/76	0.000003	0.000141	(Up) *NFKBIA, TNF, IL‐6, IL‐8, CXCL1, CXCL2, TNFAIP3, PYCARD, TCONS_00266386*
Salmonella infection	ko05132	10/107	0.000008	0.000277	(Up) *IL‐6, IL‐8, CXCL1, CXCL2, CXCL3, FOS, JUN, CSF2, PYCARD, DYNC2H1*
Legionellosis	ko05134	9/92	0.000016	0.000461	(Up) *HSPA6, IL‐6, IL‐8, CXCL1, CXCL2, CXCL3, NFKBIA, TNF, PYCARD*
Jak–STAT signaling pathway	ko04630	12/185	0.000040	0.001022	(Up) *SPRY2, SPRY4, SOCS1, IL‐6, IL‐11, IL‐24, LIF, MYC, SOCS3, CSF2, CLCF1* (Down) *SPRY1*
Signaling pathways regulating pluripotency of stem cells	ko04550	11/163	0.000060	0.001325	(Up) *ID1, ID2, ID3, ID4, LIF, MYC, TBX3, INHBA* (Down) *OTX1, INHBE, POU5F1*
MAPK signaling pathway	ko04010	15/318	0.000173	0.003396	(Up) *HSPA6, PDGFRB, BDNF, MYC, TNF, DUSP1, DUSP2, DUSP4, DUSP5, MAP2K3, JUN, FOS, NR4A1, TCONS_00118147, TCONS_00266386*
Osteoclast differentiation	ko04380	11/210	0.000554	0.009800	(Up) *SOCS1, SOCS3, NFKBIA, TNF, JUN, JUNB, FOS, FOSB, FOSL1, TCONS_00118147, TCONS_00266386*

Abbreviations: DEG, differentially expressed gene; HCMEC/D3, a type of human cerebral microvascular endothelial cells; MAPK, mitogen‐activated protein kinase; OGD/R, oxygen–glucose deprivation and recovery; STAT, signal transducers and activators of transcription; TGF, transforming growth factor; TNF, tumor necrosis factor.

GO analysis showed that for the DEGs between the OGD/R group and the OGD group, the top three biological processes were cellular processes, single‐organism processes, and response to stimuli; the top three terms related to cellular components were cell, cell part, and organelle compartments, and the top three terms related to molecular functions were binding, catalytic activity, and nucleic acid‐binding transcription factor activity (Figure S3).

### 3.2 Proteomic analysis of HCMECs subjected to OGD/R

By using a threshold of a 1.5‐FC and a *p* value of <0.05, 201 differentially expressed proteins (DEPs) between the OGD/R and OGD group were identified, including 96 upregulated and 105 downregulated proteins (Figure 1C,D; Data S4).

DEPs‐based KEGG enrichment analysis showed that the top five significantly enriched pathways were glycosaminoglycan biosynthesis‐chondroitin sulfate/dermatan sulfate, extracellular matrix (ECM)–receptor interaction, ATP‐binding cassette (ABC) transporters, *Staphylococcus aureus* infection, and vitamin digestion and absorption (Table 2; Data S5), suggesting their potential roles in reperfusion injury. Further analysis of the top ten enriched pathways between OGD/R and OGD demonstrated that pathways related to ECM destruction (evidenced by downregulation of COL4A1, LAMA4, ITGA1, ITGA7, ITGB3, and HSPG2) and remodeling (evidenced by upregulation of PIGT and THBS1) were the most significantly enriched pathways. Therefore, our results show that destruction and remodeling of the ECM is an important feature of endothelial reperfusion injury at the protein level. In addition, reperfusion injury also resulted in impaired endothelial transport functions, shown by downregulation of ABC transporters (ABCD1, ABCA3, ABCB7) and multivitamin transporter SLC5A6. Consistent to the results of RNA‐seq, proteomic analysis also revealed an inflammatory response in brain ECs after reperfusion injury, demonstrated by upregulation of proteins involved in *Staphylococcus aureus* infection and cytosolic DNA‐sensing pathway. Whole transcriptome rank‐based GSEA analysis also reveals a strong enrichment in pathways related to ECM destruction (ECM‐receptor interaction pathway) and inflammatory response (cell adhesion molecules pathway), shown in Figure S2 and Data S6.

**TABLE 2:** Top ten pathways associated with DEPs in HCMEC/D3 cells in the OGD/R group, as compared with the OGD group.

Pathway	Pathway ID	Adjusted *p* value	Enrichment score	Associated proteins
Glycosaminoglycan biosynthesis‐chondroitin sulfate / dermatan sulfate	hsa00532	0	29.677419	(Up) PIGT
ECM‐receptor interaction	hsa04512	0.000187	7.419355	(Up) THBS1 (Down) COL4A1, ITGA7, ITGB3, LAMA4, ITGA1, HSPG2
ABC transporters	hsa02010	0.004323	11.129032	(Down) ABCD1, ABCA3, ABCB7
Staphylococcus aureus infection	hsa05150	0.006148	14.838710	(Up) FGG (Down) KRT10
Vitamin digestion and absorption	hsa04977	0.010892	11.870968	(Up) APOB (Down) SLC5A6
Cytosolic DNA‐sensing pathway	hsa04623	0.010892	4.946237	(Up) POLR3H, NFKBIA, NFKBIB (Down) CGAS, POLR2L
GPI‐anchor biosynthesis	hsa00563	0.027287	14.838710	(Up) PIGT
PI3K‐Akt signaling pathway	hsa04151	0.031378	2.558398	(Up) THBS1, STK11, NR4A1 (Down) COL4A1, EIF4EBP1, ITGA7, ITGB3, PPP2R2D, LAMA4, ITGA1
Cholesterol metabolism	hsa04979	0.056043	4.685908	(Up) APOB (Down) PCSK9, SORT1
Ubiquinone and other terpenoid‐quinone biosynthesis	hsa00130	0.056043	9.892473	(Up) NQO1

Abbreviations: ABC, ATP‐binding cassette; DEP, differentially expressed protein; ECM, extracellular matrix; GPI, glycosylphosphatidylinositol; HCMEC/D3, a type of human cerebral microvascular endothelial cells; OGD/R, oxygen–glucose deprivation and recovery; PI3K, phosphatidylinositol 3′‐kinase.

We annotated 151 DEPs to 1010 GO function entries. The most enriched biological processes were cellular processes, biological regulation, metabolic processes, and regulation of biological processes; the most enriched cellular component GO terms were cell, cell part, and organelle compartments; the most enriched molecular function GO terms were binding, catalytic activity, enzyme regulator activity, and transporter activity (Figure S4).

### 3.3 Integrative analysis of the transcriptomics and proteomics data

To investigate correlations between mRNA expression and protein expression levels, we performed a combined transcriptomic and proteomic analysis of data from the OGD/R and OGD groups (Figure 1E). We generated a scatter plot of the mRNA expression levels of known genes (X‐axis) versus the corresponding protein expression levels (Y‐axis) to compare the protein versus mRNA abundances. The correlation value (*r* = 0.492), calculated using Spearman's rank correlation coefficient test, suggested little correlation between mRNA expression and protein expression (Figure S5). Integrative analysis showed that six genes (*DUSP1*, *JUNB*, *NFKBIA*, *NR4A1*, *SERPINE1*, and *THBS1*) were differentially expressed at both the mRNA and protein levels, and all of them were upregulated (Figure 1E,F; Data S7).

### 3.4 TNF signaling pathway was upregulated in the brain of mice receiving MCAO/R

**FIGURE 2:**
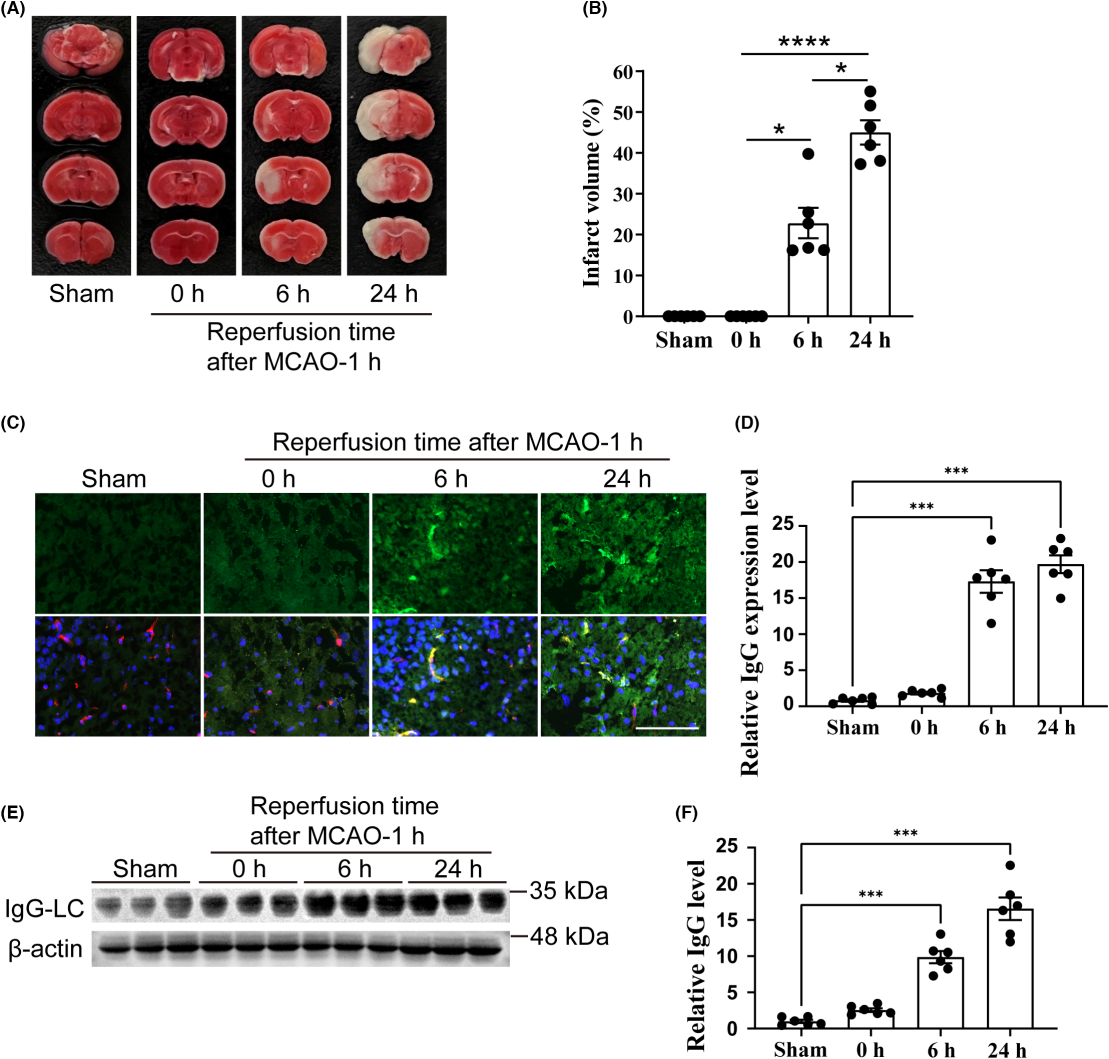
Mouse model of MCAO/R and assessment of brain microvascular injury. (A) TTC staining of mouse brain tissues after 0, 6, or 24 h of reperfusion following 1 h of MCAO induction (*n* = 6 mice per group). Infarcted brain tissues were visualized by TTC staining. (B) Quantification of infarct sizes, as determined by TTC staining. (C,D) Immunofluorescence staining and quantification of endogenous plasma IgG (green) extravasation from brain vessels (red) of mice after 0, 6, or 24 h of reperfusion following 1 h of MCAO induction or a sham operation (*n* = 6 mice per group). Scale bar, 100 μm. (E,F) Western blotting and quantification of endogenous plasma IgG extravasation in brain parenchyma of mice after 0, 6, or 24 h of reperfusion following 1 h of MCAO induction or a sham operation (*n* = 6 mice per group). *p < 0.05, ***p < 0.001, ****p < 0.0001 by one‐way ANOVA followed by Tukey's multiple‐comparisons test. LC, light chain; MCAO/R, middle cerebral artery occlusion/reperfusion; TTC, 2,3,5‐triphenyltetrazolium chloride.

In mice subjected to 1 h‐MCAO without reperfusion, infarction had not developed based on TTC staining (Figure 2A,B). Extensive infarction was detected in the cortical and striatum regions in brain tissues at both 6 h (24.0% ± 7.9%, *p* = 0.0115) and 24 h (45.5% ± 4.6%, *p* < 0.0001) after reperfusion, especially at 24 h post‐reperfusion (Figure 2A,B). As we showed previously, the growth of infarct size at 24 h post‐reperfusion also leads to increased neurological deficits.^11^ We next assessed brain microvascular injury by detecting mouse blood IgG extravasation from vessels in the ischemic and infarct areas. The results showed that blood IgG leakage increased significantly in mice after 6 and 24 h of reperfusion (*p* < 0.0001; Figure 2C–F). These results demonstrate that reperfusion following cerebral ischemia led to severe brain infarction and microvascular injury shown by BBB disruption.

**FIGURE 3:**
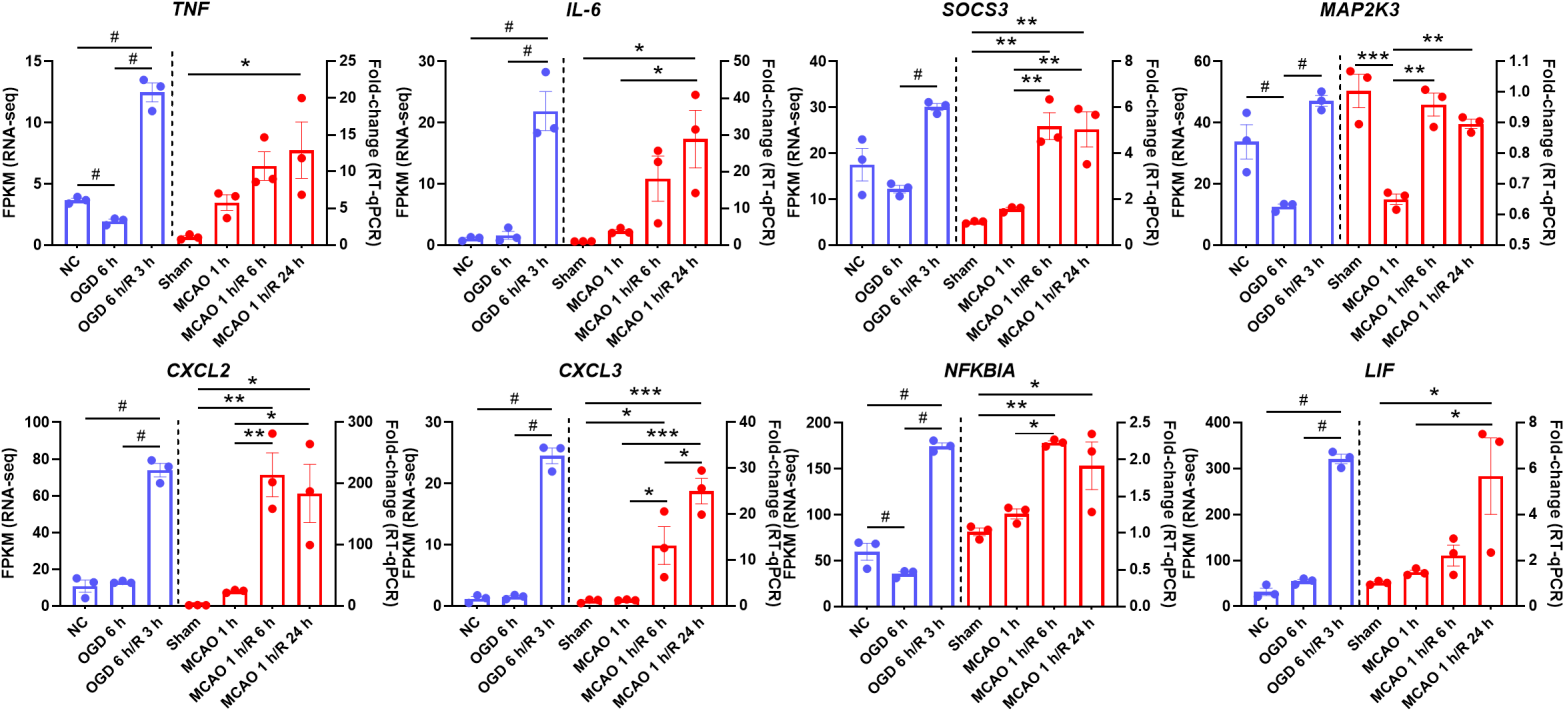
Consistent upregulation of the TNF signaling pathway in both HCMEC/D3 cells and a mouse model of MCAO/R. The left part of the histograms shows the RNA‐seq data of HCMEC/D3 cells (#*p* < 0.05, *n* = 3 biological replicates per group), and the right part of the histograms shows the relative mRNA levels in MCAO/R mouse brain tissues determined by RT‐qPCR (*p < 0.05, **p < 0.01, ***p < 0.001, ****p < 0.0001 by one‐way ANOVA followed by Tukey's multiple‐comparisons test, *n* = 3 mice per group). HCMEC/D3, a type of human cerebral microvascular endothelial cells; MCAO/R, middle cerebral artery occlusion/reperfusion; TNF, tumor necrosis factor.

Seventeen DEGs associated with the TNF signaling pathway found by RNA‐seq were selected and validated in the mouse MCAO/R model using RT‐qPCR and immunofluorescence (IF) analyses (Table 1). We selected genes in this pathway because it was the most significantly enriched pathway. We firstly quantified mRNA expression changes for the 17 DEGs of the TNF signaling pathway by RT‐qPCR. Consistent with the RNA‐seq data, RT‐qPCR data showed that the mRNA levels of most genes (*TNF*, *IL‐6*, *SOCS3*, *CXCL1*, *CXCL2*, *CXCL3*, *MAP2K3*, *NFKBIA*, *LIF*, *JUN*, *EDN1*, and *CSF2*) also showed upregulations (Figure 3; Figure S6). As further validation, the protein levels of TNF‐α, IL‐6, and CXCL3 were assessed by IF staining analysis. The results showed that these proteins were significantly upregulated after 24 h reperfusion following 1 h‐ischemia (Figure S7), which were consistent with the RT‐qPCR results.

### 3.5 Inhibiting TNF‐α significantly attenuated brain endothelial inflammation in mice following cerebral ischemia and reperfusion

**FIGURE 4:**
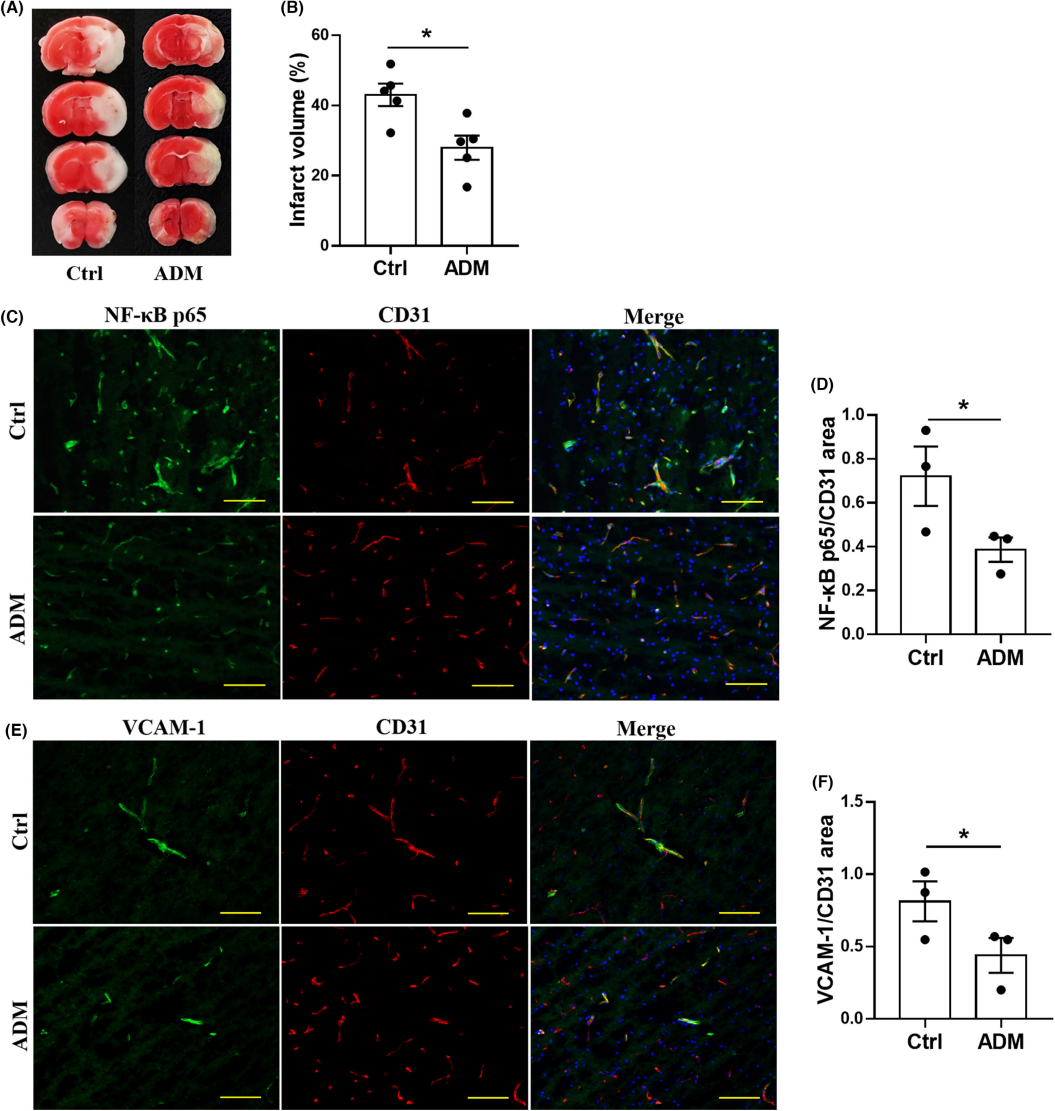
TNF mAb (adalimumab) attenuated endothelial inflammation in mice following cerebral ischemia and reperfusion. (A) TTC staining of coronal brain tissue slices after 1 h‐MCAO and 24 h reperfusion. Infarcted areas were visualized as white in TTC staining. (B) Quantifications of infarct volume as determined by TTC staining in mice treated with control human IgG, or ADM (3 mg/kg) after 1 h‐MCAO and 24 h reperfusion (*n* = 5 mice per group). (C–F) Co‐immunofluorescence staining and quantifications for endothelial NF‐κB p65 or VCAM‐1 (green) with CD31 (red) in infarcted brain tissues of mice with the treatment of control IgG or ADM (*n* = 3 mice per group). Protein fluorescence signal densities were quantified and normalized to the CD31 signal area. Scale bar, 100 μm. *p < 0.05 by unpaired *t* test. ADM, adalimumab; Ctrl, control; MCAO, middle cerebral artery occlusion.

In view of our omics results, we found that the inflammatory response was one of the main features of endothelial reperfusion injury following ischemia, and TNF pathway was one of the most enriched inflammatory signaling pathways, so we determined the effect of intravenous injection of adalimumab, a TNF‐α neutralizing antibody used in clinic, on mice with cerebral ischemia and reperfusion. We first assessed the TNF‐α downstream inflammation markers NF‐κB p65 and VCAM‐1. We found that the expression of NF‐κB p65 and VCAM‐1 in ECs increased gradually with the prolongation of reperfusion time (data not shown). Subsequently, we found that adalimumab significantly reduced the infarct volume to 28.0% ± 3.5%, compared with the control group (43.0% ± 3.2%; *t* = 3.196, *p* = 0.0127; Figure 4A,B). Further studies showed that adalimumab significantly reduced the expression of NF‐κB p65 (*t* = 2.750, *p* = 0.0205; Figure 4C,D) and VCAM‐1 (*t* = 2.390, *p* = 0.0341; Figure 4E,F) on cerebral ECs in mice with MCAO‐1 h and reperfusion‐24 h. Our results suggested that inhibition of TNF‐α is a promising therapeutic approach for reducing brain endothelial inflammation and thus microvascular reperfusion injury after acute IS.

### 3.6 THBS1 but not PIGT was upregulated in the brain ECs of mice following cerebral ischemia and reperfusion

Next, we validated the proteomics results in the mouse model of cerebral ischemia and reperfusion. We selected two genes *THBS1* (encoding Thrombospondin‐1 or THBS1) and *PIGT* (encoding GPI transamidase component PIG‐T or PIGT) for further validation, because *THBS1* was upregulated by OGD/R both at the mRNA and protein levels in vitro, and PIGT represents the most significantly enriched pathway in the proteomics analysis (Table 2). The protein levels of THBS1 and PIGT in mouse brain were assessed by both IF staining and Western blotting analysis.

**FIGURE 5:**
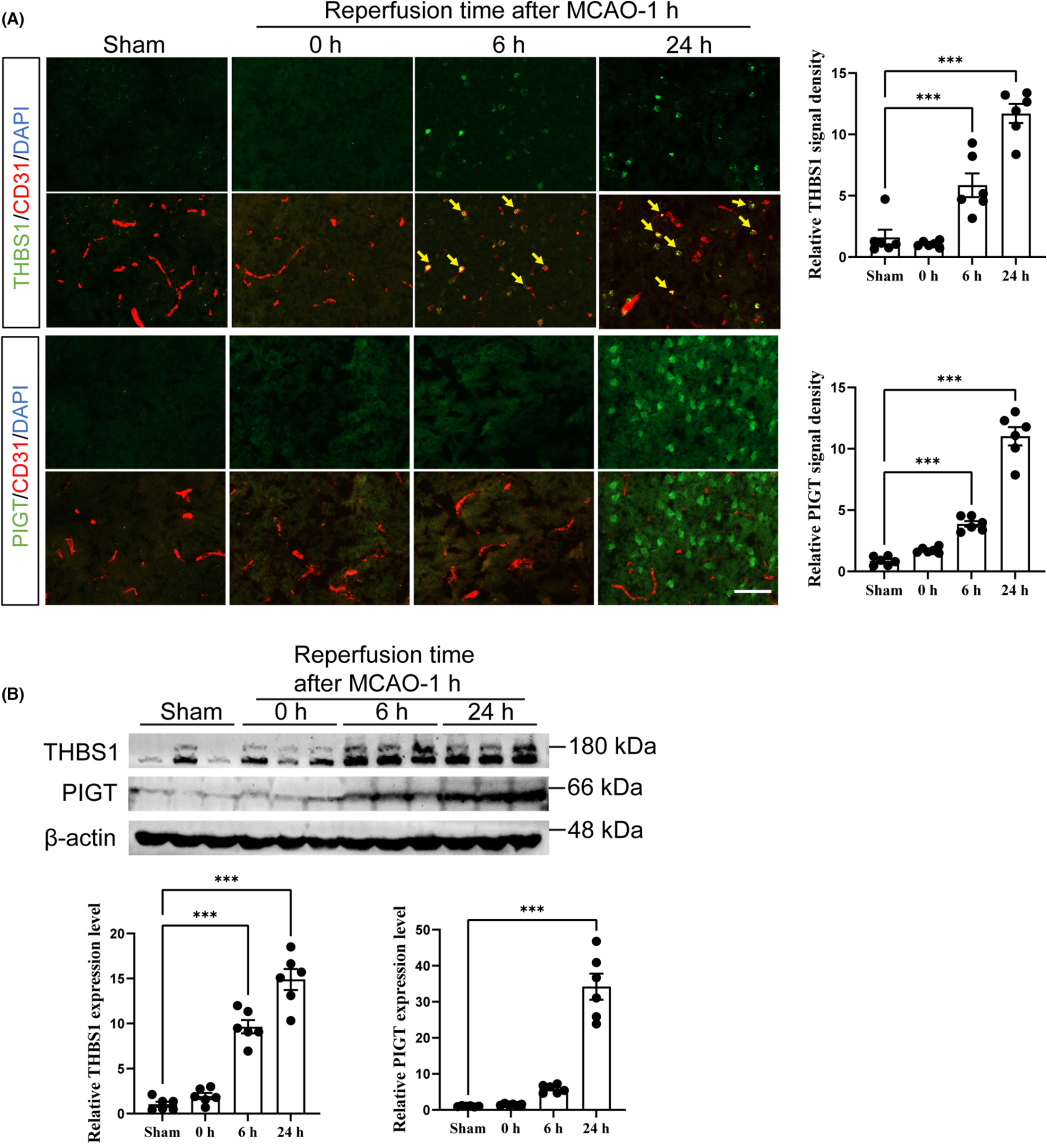
Measurement of the protein levels of THBS1 and PIGT in mice after cerebral ischemia and reperfusion. (A) Co‐immunofluorescence staining and quantification of THBS1 and PIGT (green) with the endothelial marker CD31 (red) was performed in the ipsilateral cortex of mouse brain tissues after 0, 6, or 24 h of reperfusion following 1 h of MCAO induction or a sham operation (*n* = 6 mice per group). (B) Western blotting and quantification of THBS1 and PIGT in brain parenchyma of mice after 0, 6, or 24 h of reperfusion following 1 h of MCAO induction or a sham operation (*n* = 6 mice per group). Scale bar, 50 μm. ***p < 0.001 by one‐way ANOVA followed by Tukey's multiple‐comparisons test. MCAO, middle cerebral artery occlusion.

The results showed that THBS1 was absent in sham mice or mice receiving only 1 h‐MCAO but was significantly upregulated after 6 and 24 h reperfusion following 1 h‐MCAO (Figure 5A,B), in line with the proteomics data. Consistent with THBS1 being a secreted adhesive glycoprotein that resides in ECM and cell surface,^12^ abundant THBS1 was found in both brain ECs and the surrounding area in mice receiving MCAO/R (Figure 5A). We also measured the protein level of PIGT in mouse brain tissue and found that similar to THBS1, PIGT was not detected in sham mice or mice receiving only 1 h‐MCAO and was significantly upregulated after 6 and 24 h reperfusion following 1 h‐MCAO (Figure 5A,B). However, IF staining showed that the PIGT signal was mainly located in neurons and diffusely present in brain parenchyma, without clear co‐localization with brain ECs (Figure 5A). These results demonstrate that THBS1, but not PIGT, was potentially involved in reperfusion‐related brain microvascular injury in vivo.

## 4 DISCUSSION

The mechanisms underlying reperfusion injury are complex and involve many pathological factors, such as inflammatory responses, mitochondrial dysfunction, free radical production, and Ca^2+^ overload.^13^, ^14^ Previous studies have shown that anti‐inflammatory molecules,^15^ antioxidants,^16^, ^17^ and calcium channel blockers can reduce cerebral reperfusion injury. Targeting mitochondria may also be helpful for preventing and treating reperfusion injury.^14^ However, it is worth mentioning that although many neuroprotective agents have shown efficacy in experimental cerebral ischemia/reperfusion injury, clinical studies have failed to demonstrate positive outcomes, and only a few drugs, such as edaravone and butylphthalide,^17^, ^18^ are currently in clinical use. The low success rate of drug development for acute IS may reflect the facts that the mechanisms of reperfusion injury are still not fully clear. As mentioned above, damage to ECs is the main pathological basis of reperfusion injury. Thus, gaining insight into the molecular signatures of brain ECs during reperfusion injury will aid in developing multi‐pathway and multi‐target intervention strategies against reperfusion injury.

In this study, we analyzed the transcriptomic and proteomic changes in HCMECs during reperfusion injury. Pathway analysis of the transcriptomics data showed that the TNF signaling pathway was the most significantly enriched pathway after OGD/R versus OGD, suggesting that upregulation of the pathway is an important cause of endothelial reperfusion injury. In addition, the top significantly changed signaling pathways were mostly associated with the endothelial cell‐mediated inflammatory responses, including TNF signaling pathway, TGF‐β signaling pathway, cytokine–cytokine receptor interaction, NOD‐like receptor signaling pathway, JAK–STAT signaling pathway, MAPK signaling pathway, and NF‐κB signaling pathway. Previous results showed that an excessive inflammatory response contributes to the pathogenesis of cerebral ischemia reperfusion injury^15^, ^19^ and that the inflammatory response is mainly mediated by microglia, astrocytes, and peripheral leukocytes.^20^ Our results suggest that ECs may also play an important role in mediating inflammation, especially through the TNF signaling pathway.

RT‐qPCR was performed using a mouse model of MCAO/R to quantify changes in 17 DEGs associated with the TNF signaling pathway at the mRNA level. By comparing the RT‐qPCR and RNA‐seq data, we found that most genes showed consistent expression changes. However, it must be mentioned that there are limitations in translating results from a human endothelial cell line to a mouse model as we have done in this study. Results may vary when comparing cells in vivo and in vitro because of the differences in environment and mechanisms. Protein expression levels of the TNF‐α, IL‐6, and CXCL3 genes were further verified by IF staining analysis. Results showed that these proteins were significantly upregulated after ischemia for 1 h and remained at high levels after reperfusion, which was consistent with the RT‐qPCR results, suggesting that these genes are important targets for preventing and treating reperfusion injury. Among them, IL‐6 is a proinflammatory cytokine and CXCL3 is a chemokine, but their specific roles in cerebral reperfusion injury have not been clarified. Nevertheless, our findings indicate that these genes and their associated pathways may be important for endothelial cell reperfusion injury.

Studies have indicated that dysfunctional TNF pathway signaling plays important roles in the occurrence and development of many diseases, such as inflammatory and autoimmune diseases, cancer, and cardiovascular disease.^21^, ^22^ TNF‐α is the most important molecule in the TNF signaling pathway and is considered as a major proinflammatory mediator. Interestingly, in pathological and physiological situations, TNF‐α serves dual functions.^22^ TNF‐α has been reported to be both neurotoxic and neuroprotective in nervous system diseases. Previous data generated using a rat model of MCAO/R showed that TNF‐α mRNA expression was increased in brain tissue,^23^ and in vitro OGR can enhance TNF‐α secretion from microglia.^24^ Inhibiting TNF‐α in mouse models of cerebral ischemia/reperfusion decreased cerebral infarction and improved the functional outcomes.^25^, ^26^, ^27^, ^28^ However, inhibiting other proinflammatory genes, such as NF‐κB, MAPK, and IL‐1β, can also reduce cerebral ischemia reperfusion injury in animals.^15^ Therefore, the critical inducing factors of reperfusion‐caused endothelial inflammation remain to be further identified. Here, we firstly showed that inhibition of TNF‐α with adalimumab can reduce the cerebral infarct volume. Furthermore, we showed that inhibition of TNF‐α significantly reduces the inflammatory responses in cerebral ECs, evidenced by attenuated activation of NF‐κB p65 and expression of VCAM‐1, highlighting a critical role of TNF‐α in reperfusion‐induced endothelial inflammation. The results of our study suggest that targeting inflammatory signaling pathway members, especially those in the TNF signaling pathway, may be an important way to alleviate endothelial reperfusion injury and BBB damage.

Proteomic analysis showed that proteins associated with ECM‐receptor interaction, ABC transporters, and the PI3K‐AKT signaling pathway were significantly enriched, suggesting that the associated proteins may also play important roles in reperfusion injury. Our results show that destruction and remodeling of endothelial ECM is an important feature of endothelial reperfusion injury, such as downregulation of the basement membrane proteins collagen IV, integrin, and laminin, and upregulation of PIGT and THBS1. Collagen IV, laminin, and integrin proteins represent the main components of the basement membrane, and previous studies, including ours, demonstrate that reperfusion can cause the activation of matrix metalloproteinases and degradation of basement membrane proteins.^29^, ^30^, ^31^ In addition, dysregulation of the ubiquitin‐proteasome pathway and autophagy‐lysosome pathway, two major routes for the clearance of abnormal cellular components to maintain protein homeostasis, have been reported in cerebral ischemia.^32^ Whether and how they regulate reperfusion‐induced degradation or upregulation of the above‐mentioned proteins remains to be further studied.

Our transcriptomic and proteomic data showed little correlation between the mRNA and protein expression levels. In fact, steady‐state mRNA and protein levels have been compared in numerous studies, which also showed poor correlations.^33^ Protein expression is influenced by an array of post‐transcriptional regulatory mechanisms, the correlation between protein and mRNA levels is generally modest.^34^, ^35^ Furthermore, extremely low‐abundance proteins may be undetected when they are below the detection limits of current methods. Nevertheless, integrated transcriptomic and proteomic analyses provide a new paradigm for understanding endothelial reperfusion injury, in that protein analysis portrays the current states and reflects the immediate impact of reperfusion on pre‐existing proteins of ECs, and transcriptomic analysis reflects the impact of reperfusion on the transcripts of ECs that will manifest as proteins and modulate cell functionality at subsequent timepoints.

THBS1 is one of the few genes found to be upregulated in both transcriptomic and proteomic data, and we showed that its protein level was upregulated by reperfusion in the brain ECs of mice following cerebral ischemia. THBS1 was identified as an endogenous inhibitor of angiogenesis and regulates endothelial cell adhesion, growth, motility, survival, and senescence.^36^, ^37^ The role of THBS1 has been described in IS. Focal cerebral ischemia and subsequent reperfusion led to THBS1 expression at both the mRNA and protein levels.^38^ In a permanent mouse model of MCAO, THBS1/2 double knockout mice displayed significant deficits in synaptic density and axonal sprouting, leading to impaired neurological function.^39^ In a mouse model of traumatic brain injury, THBS1 knockout significantly worsened BBB leakage and exhibited worse neurological deficits in terms of motor and cognitive functions.^40^ These data suggest that the THBS1 may have a neuro‐ and BBB‐protective effect in IS. However, information is limited regarding the role of THBS1, especially those derived from brain ECs, in microvascular reperfusion injury and further functional studies are needed. It would be interesting to induce endothelial specific deletion or overexpression of THBS1 to determine the precise role of brain endothelial cell‐derived THBS1 in reperfusion‐related microvascular and neuronal injury.

## 5 CONCLUSIONS

In this study, we identified the dysregulated genes and pathways in brain ECs during reperfusion and provided comprehensive molecular mechanism information for further functional studies. Our findings suggest that targeting the inflammatory pathways and ECM destruction may be important approaches for attenuating microvascular reperfusion injury after IS.

## AUTHOR CONTRIBUTIONS

YJ planned and designed the experiments. YJ and YC performed the animal experiments, analyzed the data, and wrote the manuscript. XT, QG, and XH performed the animal experiments. YZM, SY, MFY, MY, and CF assisted with the animal experiments and data analysis. YW commented on the manuscript. JC and ZS conceived and supervised the project, designed the experiments, interpreted the data, and wrote the manuscript.

## FUNDING INFORMATION

The work was funded by the Guangdong Province Basic and Applied Basic Research Grant (2021B1515120089 and 2021A1515220105), National Natural Science Foundation of China (32170985), National Key Research and Development Program of China (2021YFA0910000), Science Technology and Innovation Commission of Shenzhen Municipality (JCYJ20210324115800003, JCYJ20200109114608075, SGLH20180625142404672), International collaboration project of Chinese Academy of Sciences (172644KYSB20200045), President Foundation of Nanfang Hospital (2021B021), CAS‐Croucher Funding Scheme for Joint Laboratories, and Guangdong Innovation Platform of Translational Research for Cerebrovascular Diseases.

## CONFLICT OF INTEREST STATEMENT

The authors declare that there is no conflict of interest.

## Supporting information


Supplementary S1.

Supplementary S2.

Data S1.

Data S2.

Data S3.

Data S4.

Data S5.

Data S6.

Data S7.

## Data Availability

Detailed information for this study can be found in the supplementary materials. Additional data information is available upon reasonable request from the authors.
