# Supplementary material for: Integrated transcriptomic and proteomic profiling reveals the key molecular signatures of brain endothelial reperfusion injury
Source: CNS Neurosci Ther. 2023 Oct 3;30(4):e14483. doi: 10.1111/cns.14483 (PMC11017417; doi:10.1111/cns.14483)
Supplement: Supplementary file 1 — Supplementary S1. [file CNS-30-e14483-s002.docx]

**Supplementary Figures**

**Fig. S1.** Effects of different oxygen and glucose recovery times on HCMEC/D3 viability after 6 h of OGD induction.


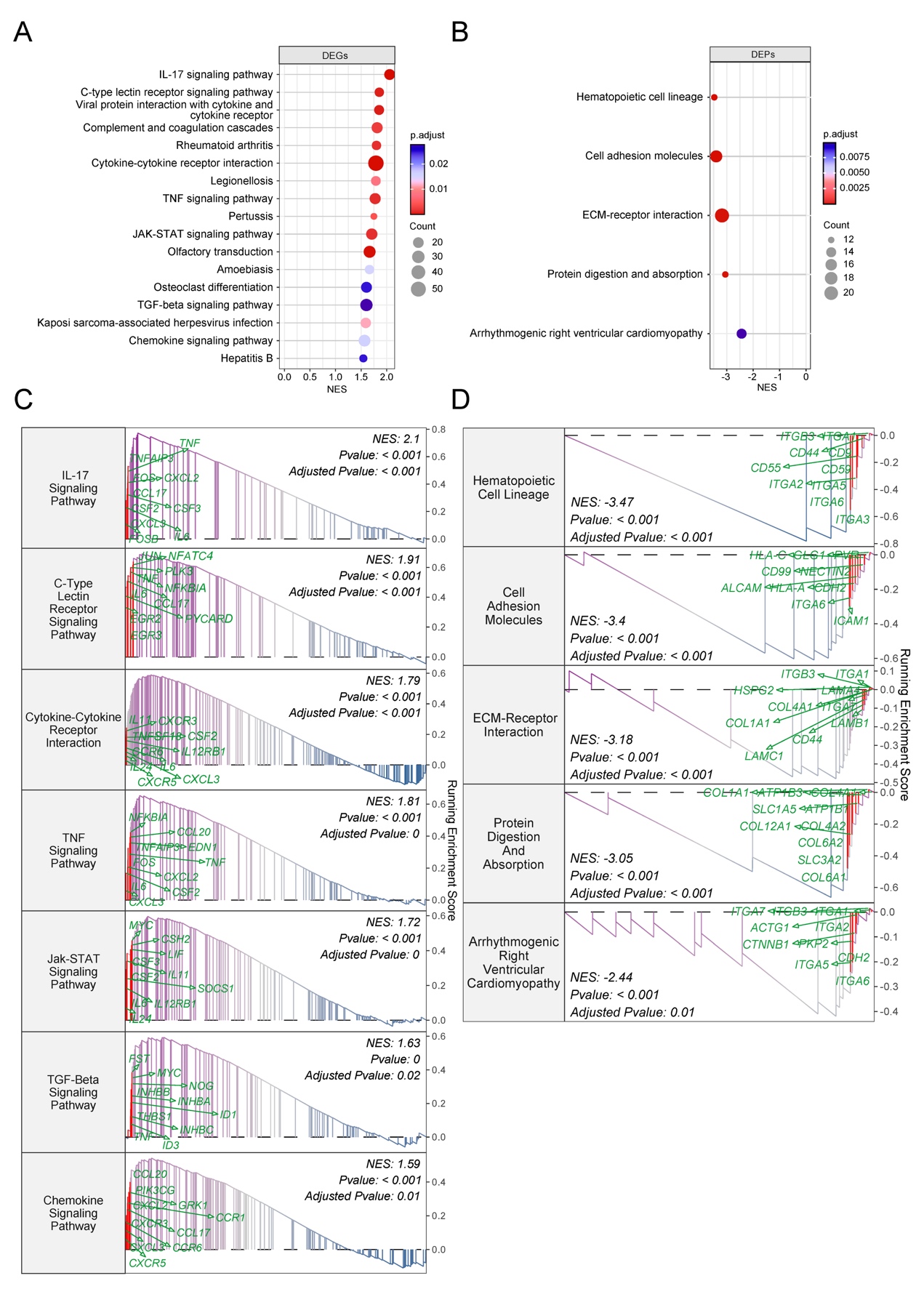


**Fig. S2.** GSEA analysis identified KEGG signaling pathways enriched at mRNAs and proteins levels. **(A)** KEGG pathway analysis of transcriptomic data using GSEA. **(B)** KEGG pathway analysis of proteomic data using GSEA. **(C)** The representative GSEA-scoring plots of transcriptomic data in OGD/R group compared with OGD group. **(D)** The representative GSEA-scoring plots of proteomic data in OGD/R group compared with OGD group. Terms were ranked based on the normalized enrichment score (NES). The top 10 leading edge genes were labelled in green. The reported NES values, p values, and adjust p values (p.adjust) were calculated with 1,000 permutations using the gseKEGG function from the R package clusterProfiler (v4.6.0).


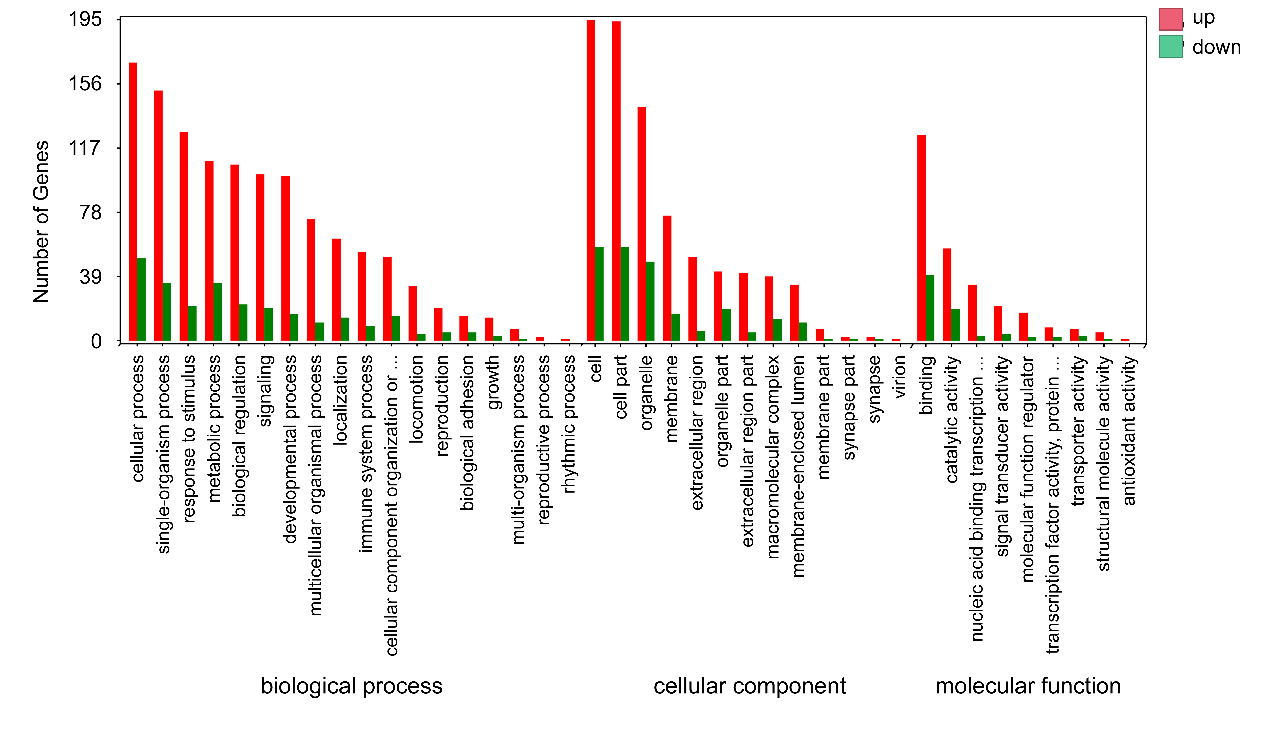


**Fig. S3.** GO term-enrichment analysis for DEGs in HCMEC/D3 cells subjected to OGD/R.


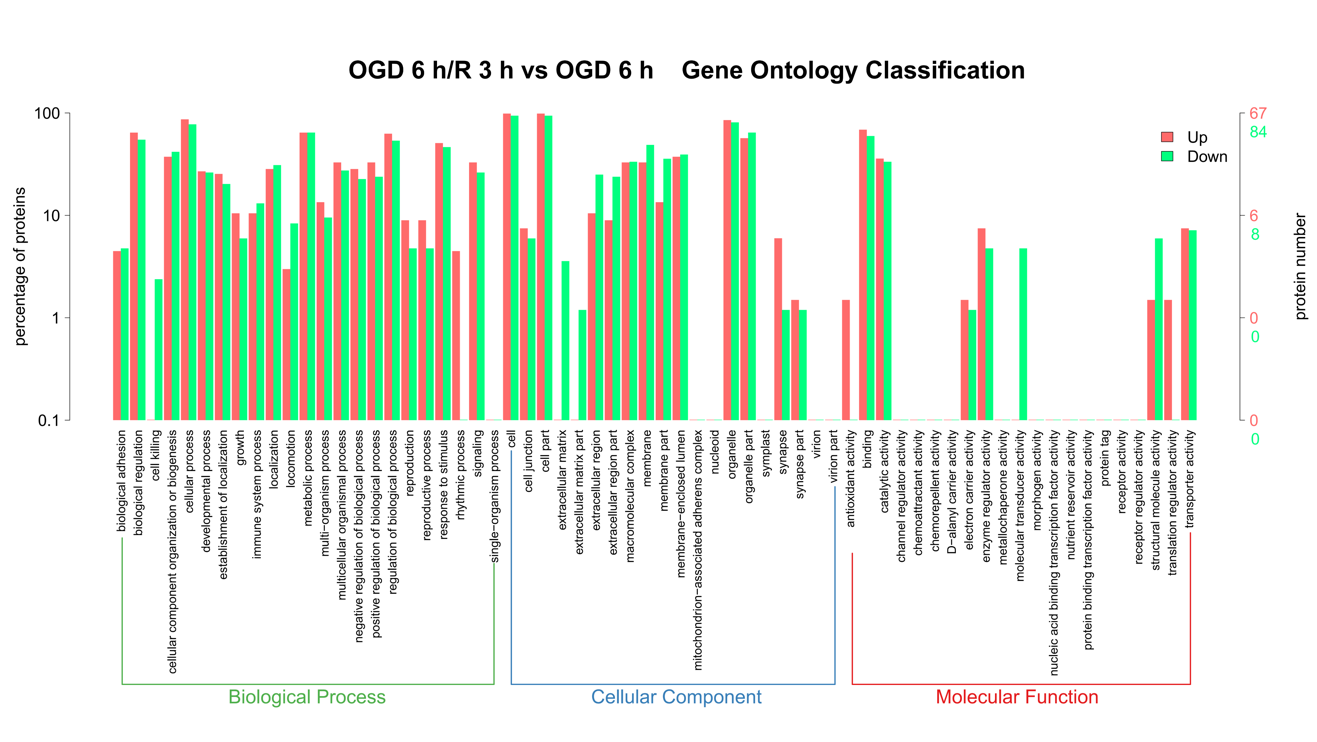


**Fig. S4.** GO term-enrichment analysis for DEPs in HCMEC/D3 cells subjected to OGD/R.


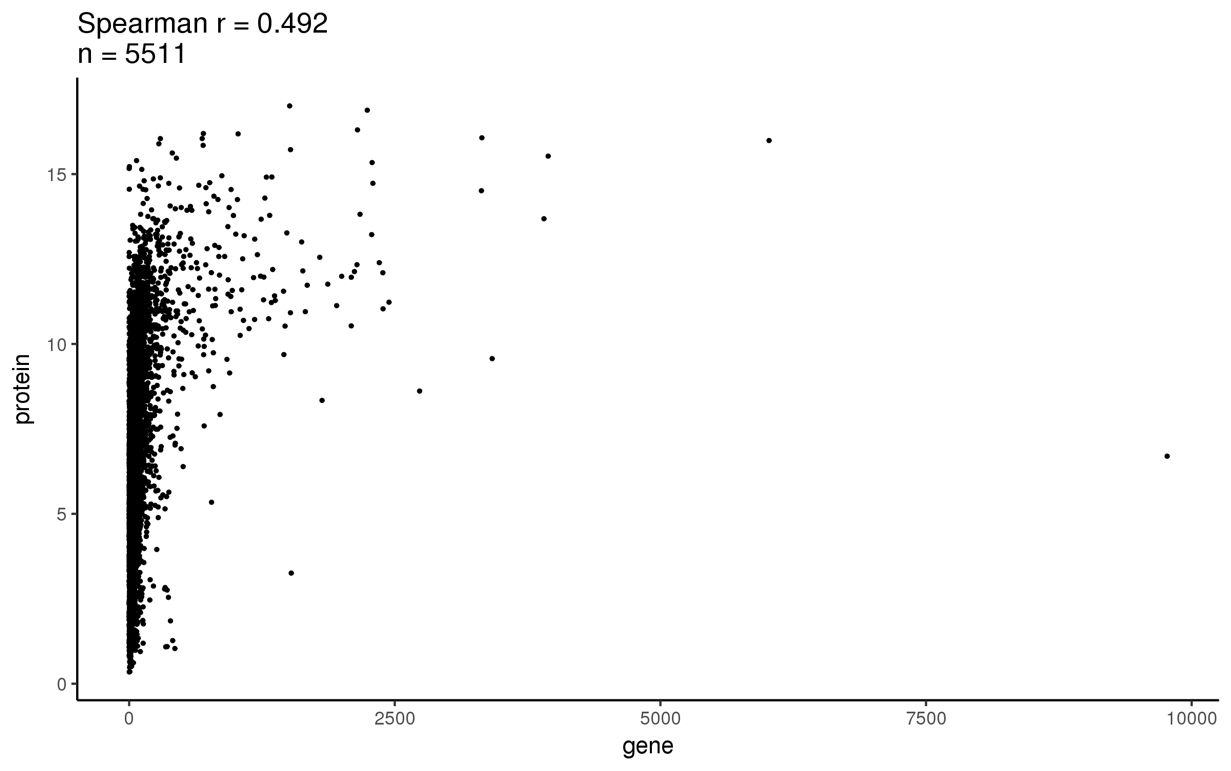


**Fig. S5.** Correlations between mRNA and protein expression levels of DEGs in HCMEC/D3 cells subjected to OGD/R.

**
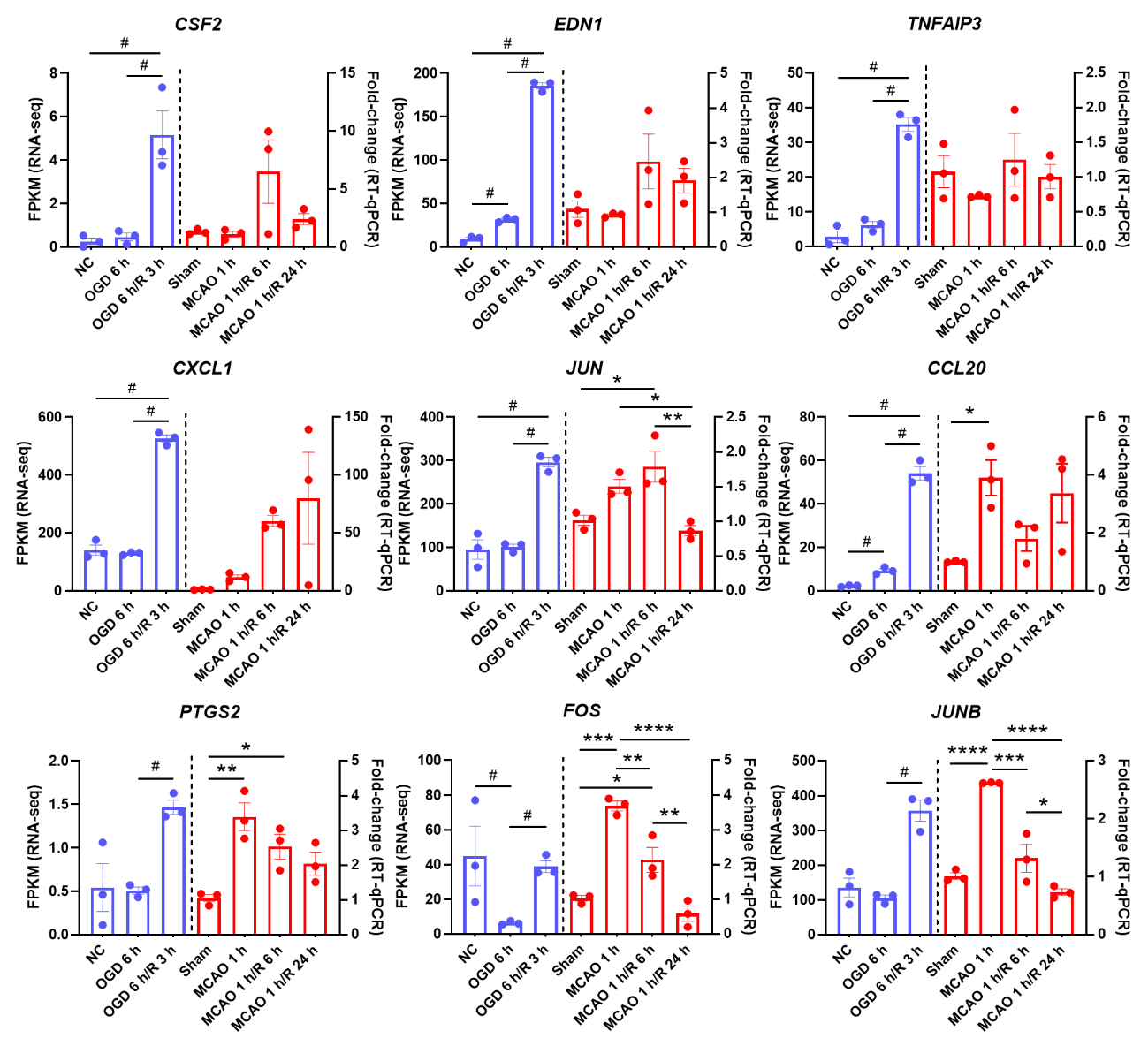
**

**Fig. S6.** Consistent upregulation of the TNF signaling pathway in both HCMEC/D3 cells and a mouse model of MCAO/R. The left part of the histograms shows the RNA-seq data of HCMEC/D3 cells (#p < 0.05, n = 3 biological replicates per group), and the right part of the histograms shows the relative mRNA levels in MCAO/R mouse brain tissues determined by RT-qPCR. *p < 0.05, **p < 0.01, ***p < 0.001, ****p < 0.0001 by one-way ANOVA followed by Tukey’s multiple-comparisons test, n = 3 mice per group.


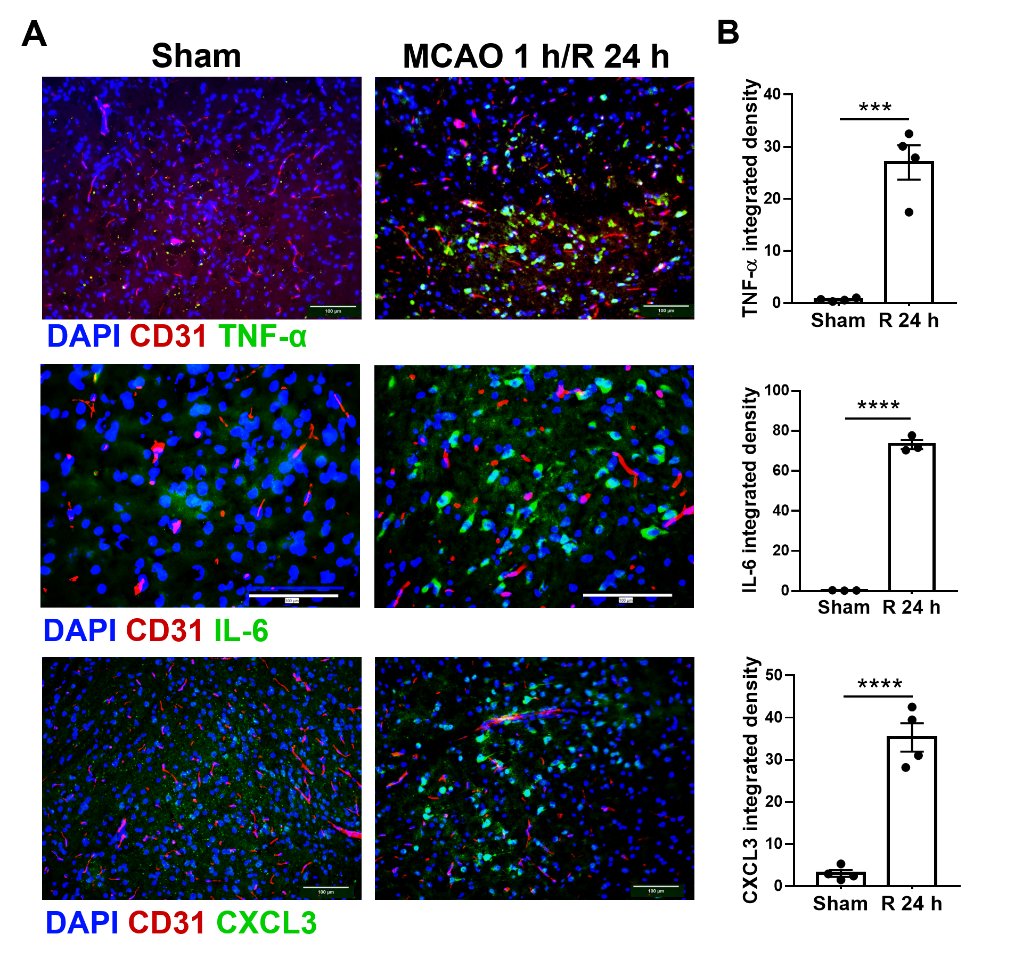


**Fig. S7.** Measurement of the protein levels of TNF signaling pathway genes in mice after cerebral ischemia and reperfusion. **(A)** Co-immunofluorescence staining for TNF-α, IL-6, and CXCL3 (green) with the endothelial marker CD31 (red) was performed in the ipsilateral cortex of mouse brain tissues after 24 h of reperfusion following 1 h of MCAO induction or a sham operation (n = 3-4 mice per group). **(B)** Quantification of the positive signals for TNF-α, IL-6, and CXCL3. Scale bar, 100 µm. ***p < 0.001 by one-way ANOVA followed by Tukey’s multiple-comparisons test. MCAO, middle cerebral artery occlusion.

**Supplementary tables**

**Table S1.** Statistical analysis of RNA-seq data from HCMEC/D3 cells subjected to OGD/R.

| **Sample** | **Clean Reads Number** | **HQ Clean Reads Number (%)** | **Read Length** | **Adapter (%)** | **Low Quality (%)** | **Poly A (%)** | **N (%)** |
| --- | --- | --- | --- | --- | --- | --- | --- |
| NC-1 | 49204132 | 48691590 (98.96%) | 150/150 | 141918 (0.38%) | 506326 (1.03%) | 2 (0%) | 0 (0%) |
| NC-2 | 48028966 | 47642254 (99.19%) | 150/150 | 106779 (0.3%) | 381030 (0.79%) | 24 (0%) | 0 (0%) |
| NC-3 | 72218078 | 71694830 (99.28%) | 150/150 | 580932 (1.07%) | 514146 (0.71%) | 192 (0%) | 0 (0%) |
| OGD-1 | 51045302 | 50631156 (99.19%) | 150/150 | 155499 (0.4%) | 407340 (0.8%) | 2 (0%) | 0 (0%) |
| OGD-2 | 47296712 | 46834206 (99.02%) | 150/150 | 132540 (0.37%) | 456256 (0.96%) | 2 (0%) | 0 (0%) |
| OGD-3 | 43954364 | 43494820 (98.95%) | 150/150 | 78804 (0.24%) | 454248 (1.03%) | 8 (0%) | 0 (0%) |
| OGD/R-1 | 51037128 | 50597596 (99.14%) | 150/150 | 137226 (0.36%) | 432642 (0.85%) | 12 (0%) | 0 (0%) |
| OGD/R-2 | 50177670 | 49796538 (99.24%) | 150/150 | 225183 (0.6%) | 374952 (0.75%) | 6 (0%) | 0 (0%) |
| OGD/R-3 | 53417286 | 53018248 (99.25%) | 150/150 | 144696 (0.36%) | 392310 (0.73%) | 2 (0%) | 0 (0%) |

HCMEC/D3, a type of human cerebral microvascular endothelial cells; OGD/R, oxygen-glucose deprivation and recovery; NC, normal control; HQ, high quality.

**Table S2.** Comparison of high-quality, clean RNA-seq data from HCMEC/D3 cells subjected to OGD/R and information deposited in the rRNA database.

| **Sample** | **All Reads Number** | **Mapped Reads** | **Unmapped Reads** |
| --- | --- | --- | --- |
| NC-1 | 24345795 | 281303 (1.16%) | 24064492 (98.84%) |
| NC-2 | 23821127 | 367692 (1.54%) | 23453435 (98.46%) |
| NC-3 | 35847415 | 1507012 (4.20%) | 34340403 (95.80%) |
| OGD-1 | 25315578 | 226407 (0.89%) | 25089171 (99.11%) |
| OGD-2 | 23417103 | 302502 (1.29%) | 23114601 (98.71%) |
| OGD-3 | 21747410 | 170897 (0.79%) | 21576513 (99.21%) |
| OGD/R-1 | 25298798 | 191815 (0.76%) | 25106983 (99.24%) |
| OGD/R-2 | 24898269 | 180938 (0.73%) | 24717331 (99.27%) |
| OGD/R-3 | 26509124 | 208213 (0.79%) | 26300911 (99.21%) |

HCMEC/D3, a type of human cerebral microvascular endothelial cells; OGD/R, oxygen-glucose deprivation and recovery; NC, normal control.

**Table S3.** Comparison of RNA-seq data of HCMEC/D3 cells treated with OGD/R with reference genome after filtering rRNA.

| **Sample** | **Total Pair Reads** | **Unmapped Pair Reads** | **Unique Mapped Pair Reads** | **Multiple Mapped Pair Reads** | **Mapping Ratio** |
| --- | --- | --- | --- | --- | --- |
| NC-1 | 48128984 | 7028873 (14.60%) | 40957765 (85.10%) | 142346 (0.30%) | 85.40% |
| NC-2 | 46906870 | 6548872 (13.96%) | 40199188 (85.70%) | 158810 (0.34%) | 86.04% |
| NC-3 | 68680806 | 9580913 (13.95%) | 58790770 (85.60%) | 309123 (0.45%) | 86.05% |
| OGD-1 | 50178342 | 7187964 (14.32%) | 42852304 (85.40%) | 138074 (0.28%) | 85.68% |
| OGD-2 | 46229202 | 6806228 (14.72%) | 39294822 (85.00%) | 128152 (0.28%) | 85.28% |
| OGD-3 | 43153026 | 6407810 (14.85%) | 36636919 (84.90%) | 108297 (0.25%) | 85.15% |
| OGD/R-1 | 50213966 | 7148981 (14.24%) | 42932941 (85.50%) | 132044 (0.26%) | 85.76% |
| OGD/R-2 | 49434662 | 6929449 (14.02%) | 42365505 (85.70%) | 139708 (0.28%) | 85.98% |
| OGD/R-3 | 52601822 | 7426185 (14.12%) | 45027160 (85.60%) | 148477 (0.28%) | 85.88% |

HCMEC/D3, a type of human cerebral microvascular endothelial cells; OGD/R, oxygen-glucose deprivation and recovery; NC, normal control.

**Table S4.** Primer sequences.

| **Primer** | **Sequence (5' to 3')** | **Base number** | **Purification way** |
| --- | --- | --- | --- |
| MU-IL6-F | TAGTCCTTCCTACCCCAATTTCC | 23 | tPAGE |
| MU-IL6-R | TTGGTCCTTAGCCACTCCTTC | 21 | tPAGE |
| MU-CSF2-F | GGCCTTGGAAGCATGTAGAGG | 21 | tPAGE |
| MU-CSF2-R | GGAGAACTCGTTAGAGACGACTT | 23 | tPAGE |
| MU-CXCL2-F | CCAACCACCAGGCTACAGG | 19 | tPAGE |
| MU-CXCL2-R | GCGTCACACTCAAGCTCTG | 19 | tPAGE |
| MU-FOS-F | CGGGTTTCAACGCCGACTA | 19 | tPAGE |
| MU-FOS-R | TTGGCACTAGAGACGGACAGA | 21 | tPAGE |
| MU-TNF-F | CCCTCACACTCAGATCATCTTCT | 23 | tPAGE |
| MU-TNF-R | GCTACGACGTGGGCTACAG | 19 | tPAGE |
| MU-TNFAIP3-F | GAACAGCGATCAGGCCAGG | 19 | tPAGE |
| MU-TNFAIP3-R | GGACAGTTGGGTGTCTCACATT | 22 | tPAGE |
| MU-EDN1-F | GCACCGGAGCTGAGAATGG | 19 | tPAGE |
| MU-EDN1-R | GTGGCAGAAGTAGACACACTC | 21 | tPAGE |
| MU-CCL20-F | GCCTCTCGTACATACAGACGC | 21 | tPAGE |
| MU-CCL20-R | CCAGTTCTGCTTTGGATCAGC | 21 | tPAGE |
| MU-LIF-F | ATTGTGCCCTTACTGCTGCTG | 21 | tPAGE |
| MU-LIF-R | GCCAGTTGATTCTTGATCTGGT | 22 | tPAGE |
| MU-CXCL1-F | CTGGGATTCACCTCAAGAACATC | 23 | tPAGE |
| MU-CXCL1-R | CAGGGTCAAGGCAAGCCTC | 19 | tPAGE |
| MU-JUNB-F | TCACGACGACTCTTACGCAG | 20 | tPAGE |
| MU-JUNB-R | CCTTGAGACCCCGATAGGGA | 20 | tPAGE |
| MU-MAP2K3-F | GCCTCAGACCAAAGGAAAATCC | 22 | tPAGE |
| MU-MAP2K3-R | GGTGTGGGGTTGGACACAG | 19 | tPAGE |
| MU-JUN-F | CCTTCTACGACGATGCCCTC | 20 | tPAGE |
| MU-JUN-R | GGTTCAAGGTCATGCTCTGTTT | 22 | tPAGE |
| MU-PTGS2-F | TTCAACACACTCTATCACTGGC | 22 | tPAGE |
| MU-PTGS2-R | AGAAGCGTTTGCGGTACTCAT | 21 | tPAGE |
| MU-SOCS3-F | ATGGTCACCCACAGCAAGTTT | 21 | tPAGE |
| MU-SOCS3-R | TCCAGTAGAATCCGCTCTCCT | 21 | tPAGE |
| MU-NFKBIA-F | TGAAGGACGAGGAGTACGAGC | 21 | tPAGE |
| MU-NFKBIA-R | TTCGTGGATGATTGCCAAGTG | 21 | tPAGE |
| MU-CXCL3-F1 | CCAACGGTGTCTGGATGTGT | 20 | tPAGE |
| MU-CXCL3-R1 | TGGCCAGCCAAGGAATACTG | 20 | tPAGE |
| MU-CXCL3-F2 | CATCCAGAGCTTGACGGTGA | 20 | tPAGE |
| MU-CXCL3-R2 | CAGACACCGTTGGGATGGAT | 20 | tPAGE |

**Table S5.** DEGs associated with the TNF signaling pathway in HCMEC/D3 cells subjected to OGD/R, compared with those subjected to OGD.

| **Gene** | **log_2_(FC)** | **P-value** | **FDR** | **Significant** |
| --- | --- | --- | --- | --- |
| CXCL3 | 4.296849222 | 2.61E-91 | 1.72E-88 | up |
| IL6 | 4.203388065 | 2.7E-89 | 1.71E-86 | up |
| CSF2 | 3.618732571 | 8.69E-15 | 1.09E-12 | up |
| CXCL2 | 3.065759735 | 6.28E-106 | 5.57E-103 | up |
| FOS | 2.969268924 | 2.12E-112 | 2.22E-109 | up |
| TNF | 2.736329188 | 1.13E-55 | 4.37E-53 | up |
| TNFAIP3 | 2.69170005 | 3.82E-113 | 4.23E-110 | up |
| EDN1 | 2.625896433 | 2.25E-125 | 3.33E-122 | up |
| CCL20 | 2.624057749 | 1.31E-62 | 5.83E-60 | up |
| NFKBIA | 2.29417942 | 6.69E-106 | 5.65E-103 | up |
| LIF | 2.28055867 | 3.12E-110 | 3.07E-107 | up |
| CXCL1 | 2.100217221 | 1.52E-99 | 1.18E-96 | up |
| JUNB | 1.821775557 | 1.6E-41 | 4.99E-39 | up |
| MAP2K3 | 1.749559054 | 9.04E-41 | 2.76E-38 | up |
| JUN | 1.608891939 | 3.58E-49 | 1.22E-46 | up |
| PTGS2 | 1.57587515 | 7.23E-17 | 1.04E-14 | up |
| SOCS3 | 1.356781523 | 1.37E-31 | 3.34E-29 | up |

HCMEC/D3, a type of human cerebral microvascular endothelial cells; OGD/R, oxygen-glucose deprivation and recovery; DEG, differentially expressed gene; TNF, tumor necrosis factor.
