# Supplementary material for: Integrated transcriptomic and proteomic profiling reveals the key molecular signatures of brain endothelial reperfusion injury
Source: CNS Neurosci Ther. 2023 Oct 3;30(4):e14483. doi: 10.1111/cns.14483 (PMC11017417; doi:10.1111/cns.14483)

Full unedited gel/blot for Figure 2

IgG (light chain, LC)

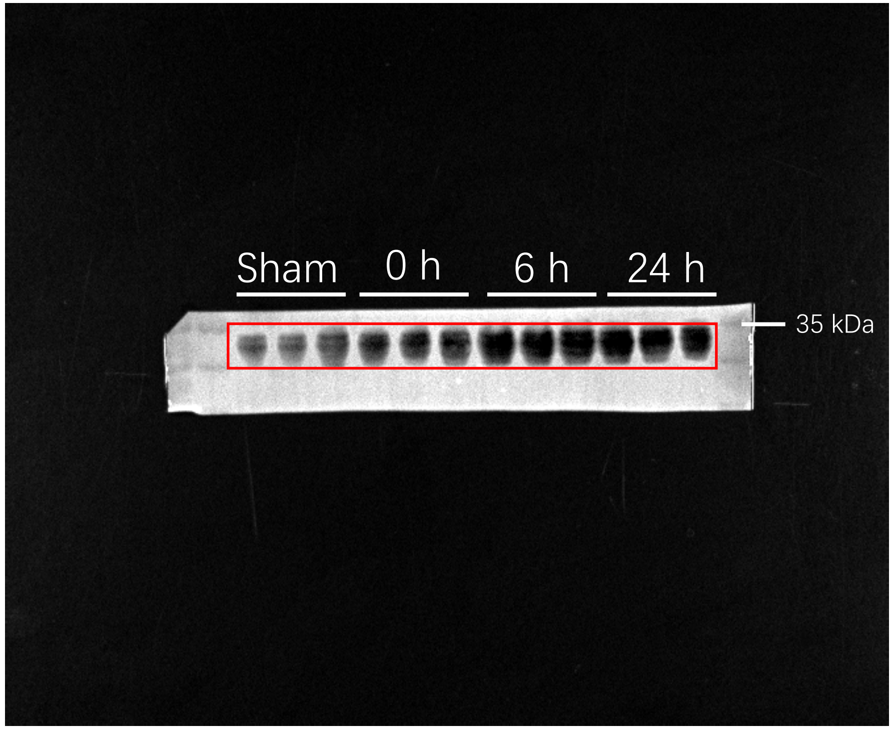

β-actin

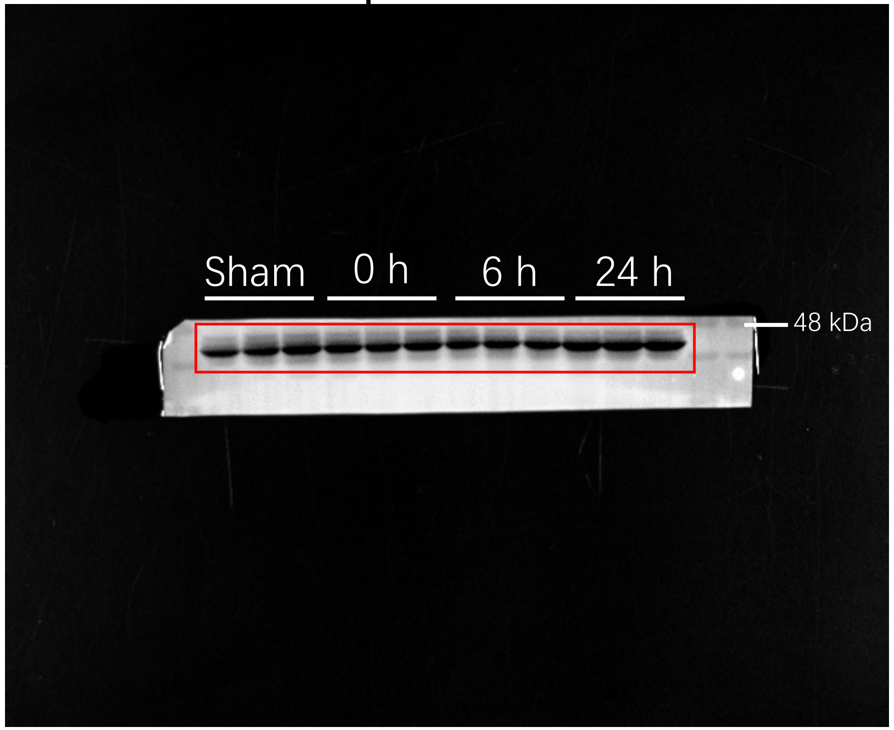

Full unedited gel/blot for Figure 5

Thrombospondin 1

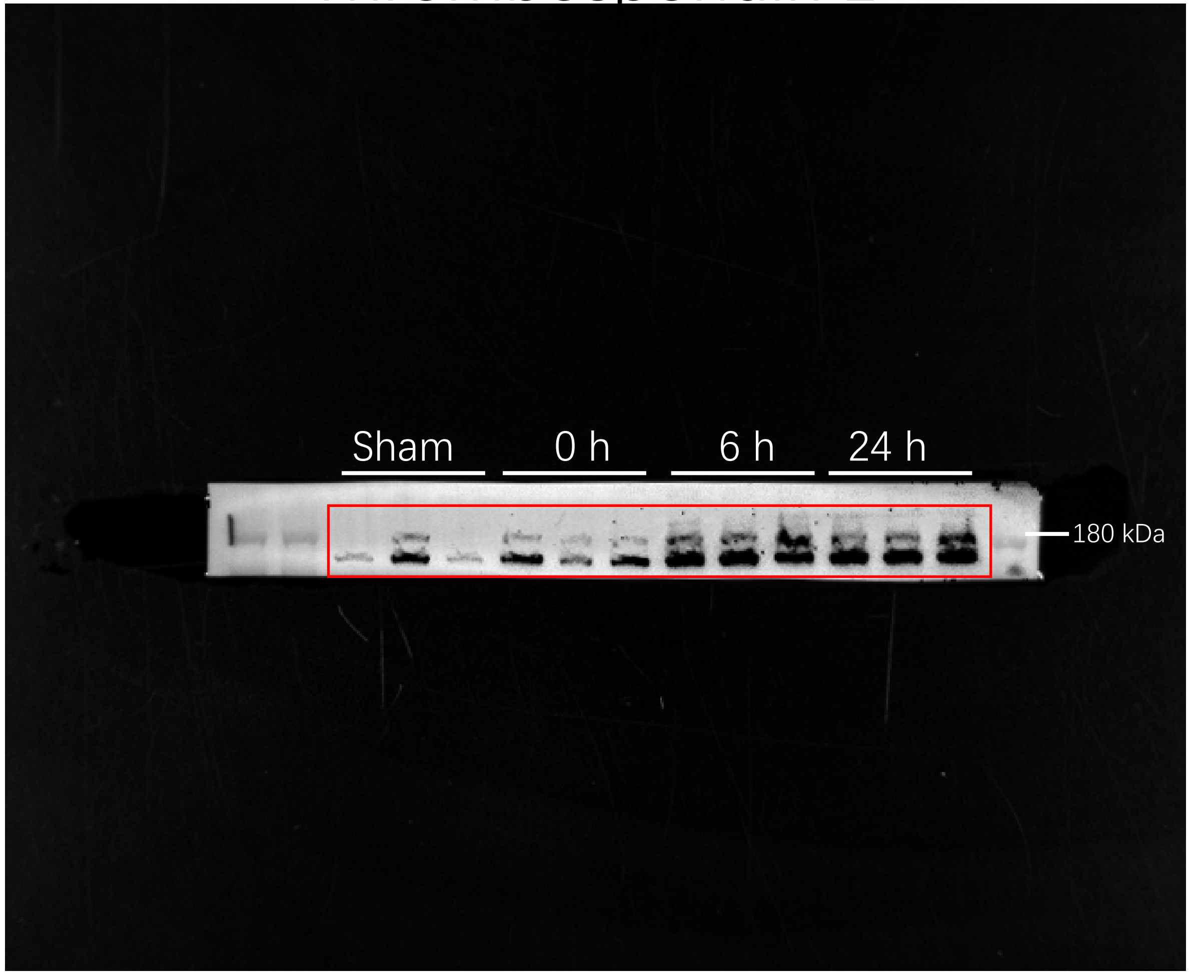

PIGT

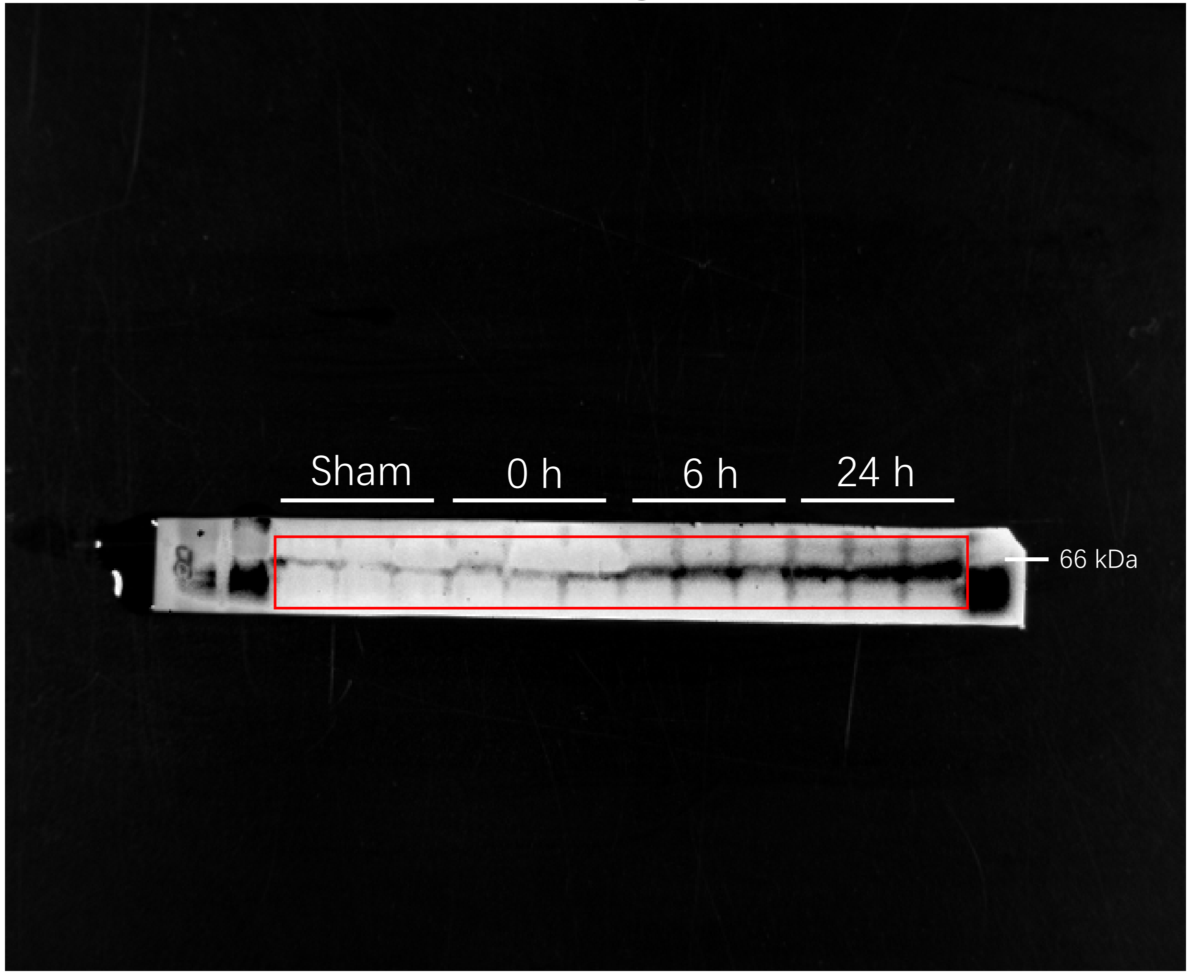

Full unedited gel/blot for Figure 5

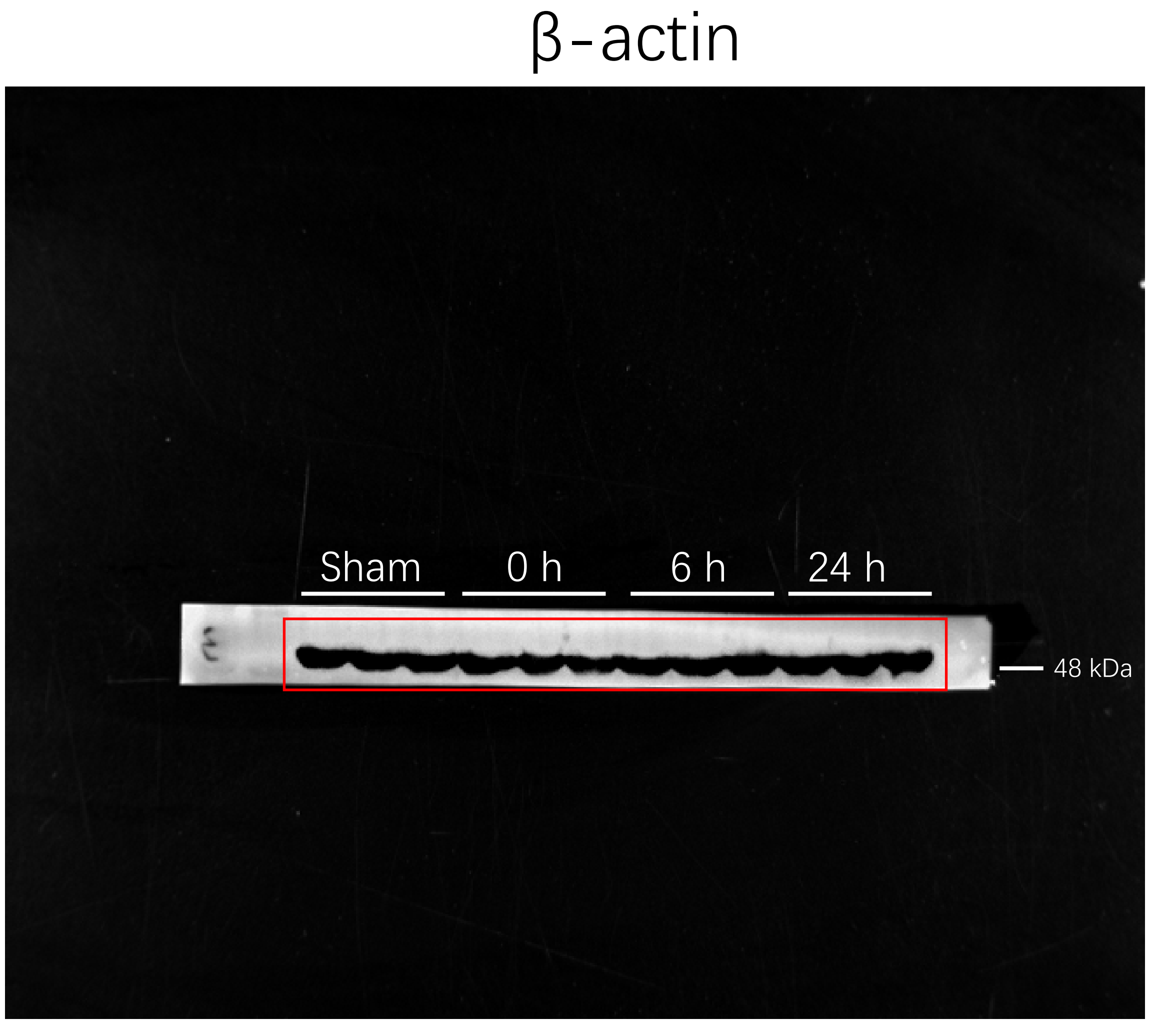

Supplement: Supplementary file 2 — Supplementary S2. [file CNS-30-e14483-s009.pdf]
